# Clenbuterol exerts antidiabetic activity through metabolic reprogramming of skeletal muscle cells

**DOI:** 10.1038/s41467-021-27540-w

**Published:** 2022-01-10

**Authors:** Jaroslawna Meister, Derek B. J. Bone, Jonas R. Knudsen, Luiz F. Barella, Thomas J. Velenosi, Dmitry Akhmedov, Regina J. Lee, Amanda H. Cohen, Oksana Gavrilova, Yinghong Cui, Gerard Karsenty, Min Chen, Lee S. Weinstein, Maximilian Kleinert, Rebecca Berdeaux, Thomas E. Jensen, Erik A. Richter, Jürgen Wess

**Affiliations:** 1 Molecular Signaling Section, Laboratory of Bioorganic Chemistry, National Institute of Diabetes and Digestive and Kidney Diseases, Bethesda, MD 20892 USA; 2 Departments of Nutrition, Exercise and Sports, University of Copenhagen, København, Denmark; 3 Faculty of Pharmaceutical Sciences, University of British Columbia, Vancouver, BC Canada; 4 Departments of Integrative Biology and Pharmacology, Houston Medical School, Houston, TX 77030 USA; 5 Mouse Metabolism Core, National Institute of Diabetes and Digestive and Kidney Diseases, Bethesda, MD 20892 USA; 6 Departments of Genetics and Development, Vagelos College of Physicians and Surgeons, Columbia University, New York, NY USA; 7 Metabolic Diseases Branch, National Institute of Diabetes and Digestive and Kidney Diseases, Bethesda, MD 20892 USA; 8 Muscle Physiology and Metabolism Group, German Institute of Human Nutrition, Potsdam-Rehbrücke, Nuthetal, Germany

**Keywords:** Diabetes, Skeletal muscle, Metabolomics, Metabolic disorders

## Abstract

Activation of the sympathetic nervous system causes pronounced metabolic changes that are mediated by multiple adrenergic receptor subtypes. Systemic treatment with β_2-_adrenergic receptor agonists results in multiple beneficial metabolic effects, including improved glucose homeostasis. To elucidate the underlying cellular and molecular mechanisms, we chronically treated wild-type mice and several newly developed mutant mouse strains with clenbuterol, a selective β_2_-adrenergic receptor agonist. Clenbuterol administration caused pronounced improvements in glucose homeostasis and prevented the metabolic deficits in mouse models of β-cell dysfunction and insulin resistance. Studies with skeletal muscle-specific mutant mice demonstrated that these metabolic improvements required activation of skeletal muscle β_2_-adrenergic receptors and the stimulatory G protein, G_s_. Unbiased transcriptomic and metabolomic analyses showed that chronic β_2_-adrenergic receptor stimulation caused metabolic reprogramming of skeletal muscle characterized by enhanced glucose utilization. These findings strongly suggest that agents targeting skeletal muscle metabolism by modulating β_2_-adrenergic receptor-dependent signaling pathways may prove beneficial as antidiabetic drugs.

## Abstract (web summary)

In this study, the authors demonstrated that agents targeting skeletal muscle metabolism by modulating β_2_-adrenergic receptor-dependent signaling may prove beneficial as novel antidiabetic drugs.

## Introduction

The number of people suffering from type 2 diabetes (T2D) and impaired glucose tolerance, a major risk factor for the development of T2D, continues to rise in an alarming manner worldwide (IDF Diabetes Atlas, 9th edn. Brussels, Belgium: 2019). Current non-insulin therapies of T2D focus mostly on targeting β-cell dysfunction (GLP-1 mimetics, DPP-4 inhibitors, sulfonylureas, and meglitinides), hepatic and adipose tissue insulin resistance (metformin and thiazolidinediones, respectively), and elimination of excess glucose through the kidneys or gut (SGLT2 and α-glucosidase inhibitors, respectively)^1^.

At present, antidiabetic drugs that directly target skeletal muscle (SKM) are not available, even though SKM is responsible for 80–90% of glucose disposal during a euglycemic-hyperinsulinemic clamp in healthy individuals^2^. Moreover, SKM insulin resistance is a key metabolic defect in the pathogenesis of T2D^2^. For these reasons, SKM is a very attractive target for the development of novel classes of antidiabetic drugs. G protein-coupled receptors (GPCRs) represent a highly druggable class of cell surface proteins. In fact, ~35% of all approved pharmaceuticals target specific GPCRs^3^. SKM expresses dozens of different GPCRs endowed with distinct G protein-coupling properties^4^. In vitro studies have shown that activation of G_q_-coupled GPCRs can regulate glucose uptake into SKM cells^5–7^. By using DREADD (Designer Receptor Exclusively Activated by a Designer Drug) technology, we recently demonstrated that receptor-mediated activation of G_q_ signaling in SKM tissue in vivo leads to greatly improved glucose tolerance^8^.

Clenbuterol is a synthetic, highly selective β_2_-adrenergic receptor (β_2_-AR) agonist that is approved for the treatment of asthma and chronic obstructive pulmonary disease in several European and Asian countries (http://drugapprovalsint.com/clenbuterol/). Similar to the β_1_- and β_3_-AR subtypes, the β_2_-AR primarily activates the stimulatory G protein G_s_, leading to increased intracellular cAMP levels and the activation of protein kinase A(PKA) and other cAMP-dependent effector proteins^9^. However, several studies have shown that β_2_-AR can also modulate cellular signaling via stimulation of cAMP inhibitory G_i_-type G proteins^10–12^. Moreover, activated β_2_-ARs, such as other GPCRs, can recruit β-arrestins (β-arrestin-1 and -2), which can also act as signaling proteins^13–15^.

β_2_-ARs are expressed at high levels in SKM, and clenbuterol and other β_2_-AR agonists have been reported to prevent or reverse muscle wasting or weakness by promoting SKM growth^16,17^. Interestingly, treatment of rodents with clenbuterol has been shown to improve glucose homeostasis^18–20^. However, the β_2_-AR is not only expressed in SKM, but also in many metabolically important tissues, including pancreatic islets and adipose tissues^21,22^. For these reasons, it remains unclear which tissues or cell types, and which cellular signaling pathways are involved in mediating the metabolically beneficial actions of clenbuterol in vivo. Specifically, the role of β_2_-ARs in regulating SKM glucose fluxes remains controversial. Although some studies reported β_2_-AR-mediated inhibition of glucose uptake into SKM^23–26^, other studies reported the opposite effect (stimulation of glucose uptake)^18,20,24,27^.

In this study, by analyzing several newly developed mouse models, we demonstrate that the beneficial metabolic effects of chronic clenbuterol are mediated by activation of SKM β_2_-ARs, and that the coupling of these receptors to SKM G_s_ is essential for the manifestation of these clenbuterol effects. In agreement with these findings, selective activation of SKM G_s_ signaling by a G_s_-coupled designer GPCR caused similar improvements in glucose homeostasis. Moreover, unbiased metabolomic and RNA-sequencing (RNA-Seq) studies shed light on the molecular mechanisms underlying the clenbuterol-induced improvements in SKM glucose homeostasis following β_2_-AR stimulation. These findings are of considerable relevance for the development of novel antidiabetic drugs targeting SKM glucose metabolism.

## Results

### Clenbuterol treatment improves whole-body glucose homeostasis in healthy lean mice and mouse models of β-cell dysfunction and insulin resistance

**Fig. 1:**
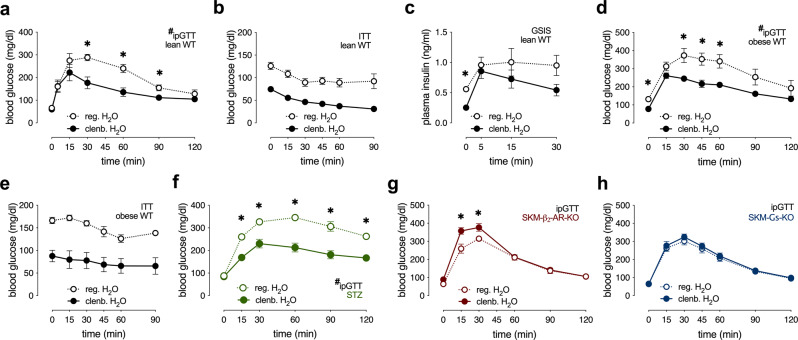
Chronic clenbuterol administration improves glucose tolerance in mice in a SKM-β_2_-AR-dependent manner. Mice consumed drinking water containing clenbuterol (30 mg/l; clenb. H_2_O) or regular drinking water (reg. H_2_O) for 5 days, followed by metabolic testing. All studies were carried out with male mice that were at least 8 weeks old. Where indicated, mice were fed a HFD for at least 8 weeks. **a** Glucose tolerance test (GTT), lean WT mice (overnight fasted) injected i.p. with glucose (2 g/kg) (*n* = 4 on reg. H_2_O, *n* = 5 on clenb. H_2_O). *p < 0.05, Student’s *t*-test, #*p* < 0.05, repeated-measure two-way ANOVA, clenbuterol effect. **b** Insulin tolerance test (ITT), lean WT mice (fasted for 4 h) injected i.p. with insulin (0.75 IU/kg) (*n* = 4 on reg. H_2_O, *n* = 5 on clenb. H_2_O). **c** Glucose-stimulated insulin secretion (GSIS), lean WT mice (overnight fasted) injected i.p. with glucose (2 g/kg) (*n* = 4 on reg. H_2_O, *n* = 5 on clenb. H_2_O). **d** GTT, HFD-induced obese WT mice (overnight fasted) injected i.p. with glucose (1 g/kg) (*n* = 7 on reg. H_2_O, *n* = 8 on clenb. H_2_O). *p < 0.05, Student’s *t*-test, #*p* < 0.05, repeated-measure two-way ANOVA, clenbuterol effect. **e** ITT, HFD-induced obese WT mice (fasted for 4 h) injected i.p. with insulin (0.75 IU/kg) (n = 7/group). **f** GTT, streptozotocin (STZ)-induced diabetic WT mice (overnight fasted) injected i.p. with glucose (1 g/kg) (*n* = 5/group). *p < 0.05, Student’s *t*-test, #*p* < 0.05, repeated-measure two-way ANOVA, clenbuterol effect. **g** GTT, lean SKM-β_2_-AR-KO mice (overnight fasted) injected i.p. with glucose (2 g/kg) (*n* = 7 on reg. H_2_O, *n* = 6 on clenb. H_2_O). **h** GTT, lean SKM-Gs-KO mice (overnight fasted) injected i.p. with glucose (2 g/kg) (*n* = 10/group). Data are presented as means ± SEM. *p < 0.05 (unpaired two-tailed Student’s *t*-test). Source data are provided as a Source data file.

To study the metabolic consequences of chronic stimulation of β_2_-ARs in vivo, we administered clenbuterol in drinking water (30 mg/l) to wild-type (WT) mice for 5 days, followed by a series of metabolic tests. Clenbuterol treatment of lean male and female WT mice led to improved glucose tolerance (Fig. 1a and Supplementary Fig. 1a). During an insulin tolerance test (ITT), lean WT mice treated with clenbuterol showed lower blood glucose levels (Fig. 1b). However, when taking into account the significantly lower basal blood glucose levels in the clenbuterol-treated mice, the insulin-mediated decrease in blood glucose levels was similar to that in the control group (Fig. 1b and Supplementary Fig. 1b). Fasting insulin levels were significantly lower following clenbuterol administration, whereas glucose-stimulated insulin secretion (GSIS) remained essentially unchanged (Fig. 1c). Importantly, clenbuterol treatment lowered blood glucose levels and improved glucose tolerance in high-fat diet (HFD)-induced obese mice (Fig. 1d, e) without improving insulin sensitivity (Fig. 1e and Supplementary Fig. 1c).

To mimic the loss of β-cell mass characteristic for advanced β-cell dysfunction, we used a streptozotocin (STZ) treatment protocol known to destroy about 70–80% of mouse β-cells^28^. Specifically, lean WT mice were injected with STZ (50 mg/kg intraperitoneally (i.p.)) for 5 consecutive days. Seven days after the last STZ injection, the mice developed characteristic signs of β-cell dysfunction including elevated fasting and fed blood glucose levels (Supplementary Fig. 1d), decreased plasma insulin concentrations (Supplementary Fig. 1e), reduced body weight (Supplementary Fig. 1f), and impaired glucose tolerance (Supplementary Fig. 1g).

Following 5-day clenbuterol administration, STZ-treated mice showed similar metabolic improvements (Fig. 1f and Supplementary Fig. 1h–j) as seen with lean and obese WT mice (Fig. 1a–e). As our data suggested that altered release of insulin does not contribute to the beneficial metabolic effects of clenbuterol treatment (Fig. 1c and Supplementary Fig. 1j) and β_2_-ARs are not expressed in adult mouse hepatocytes^29^, we speculated that the beneficial effects of clenbuterol on glucose homeostasis (Fig. 1a, d, f) were caused by activation of SKM G_s_ signaling through SKM β_2_-ARs. To test this hypothesis, we generated mice that lacked β_2_-ARs or Gα_s_ selectively in SKM (SKM-β_2_-AR-KO and SKM-Gs-KO mice, respectively). The SKM-selective deletion of the β_2_-AR and Gα_s_ was induced in adult mice via tamoxifen-dependent activation of Cre recombinase in SKM only. Efficient deletion of β_2_-AR and Gα_s_ in SKM was confirmed via quantitative real-time PCR (qRT-PCR) or western blotting, respectively (Supplementary Fig. 1k, p). The remaining SKM expression of β_2_-ARs and Gα_s_ displayed by the knockout (KO) mice may be due to the fact that SKM tissue does not only consist of myocytes but also harbors several other cell types^30^. The lack of SKM β_2_-ARs in lean mice did not lead to any obvious metabolic phenotypes (Supplementary Fig. 1l–o). In contrast, lean SKM-Gs-KO showed a significant impairment in glucose tolerance (but normal insulin sensitivity), as compared to their control littermates (Supplementary Fig. 1q–t).

Next, we treated SKM-β_2_-AR-KO and SKM-Gs-KO mice for 5 days with clenbuterol (via the drinking water). Remarkably, in the absence of SKM β_2_-ARs or SKM Gα_s_, the ability of clenbuterol treatment to improve glucose tolerance was abolished (Fig. 1g, h). These data clearly indicate that clenbuterol administration improves whole-body glucose homeostasis by stimulation of SKM β_2_-ARs and subsequent activation of SKM G_s_.

Multiple studies have shown that chronic administration of clenbuterol over a period of weeks increases SKM mass in various species^16^, raising the possibility that changes in SKM growth or mass may contribute to the beneficial metabolic effects of clenbuterol. To examine whether our 5-day clenbuterol treatment protocol resulted in an increased SKM mass, we studied mouse body composition using EchoMRI. We found that body weight (Supplementary Fig. 2a), lean mass (Supplementary Fig. 2b), and fat mass (Supplementary Fig. 2c) were not significantly affected by our clenbuterol treatment protocol. Moreover, the clenbuterol-induced improvement in glucose tolerance could already be observed as early as 36 h after initiation of clenbuterol administration (Supplementary Fig. 2d), whereas insulin secretion was unaltered (Supplementary Fig. 2e). These results suggest that the metabolic improvements of whole-body glucose homeostasis observed after several days of clenbuterol treatment are independent of changes in SKM mass.

### Chronic CNO stimulation of SKM G_s_ DREADD mice (GsD mice) mimics the metabolic improvements seen with clenbuterol-treated WT mice

As β_2_-ARs are widely expressed, we took advantage of DREADD technology to clearly define the role of chronic SKM G_s_ stimulation in regulating glucose homeostasis. DREADDs are mutant GPCRs that are insensitive to any endogenous ligands, but are selectively activated by otherwise pharmacologically inert synthetic agents, such as clozapine-N-oxide (CNO). During the past decade, DREADDs have been successfully used to study cell type-specific metabolic consequences of activating different functional classes of G proteins^31^.

To explore the in vivo metabolic effects caused by chronic stimulation of a G_s_-linked GPCR in SKM, we generated mice that expressed a G_s_-coupled DREADD (GsD)^32–36^ under the transcriptional control of the SKM-specific human α-skeletal actin (HSA) promoter. We refer to these mice from hereon simply as SKM-GsD mice. In the absence of CNO, male and female SKM-GsD mice did not show any significant changes in glucose homeostasis, as compared to their control littermates (Supplementary Fig. 3a–f).

**Fig. 2:**
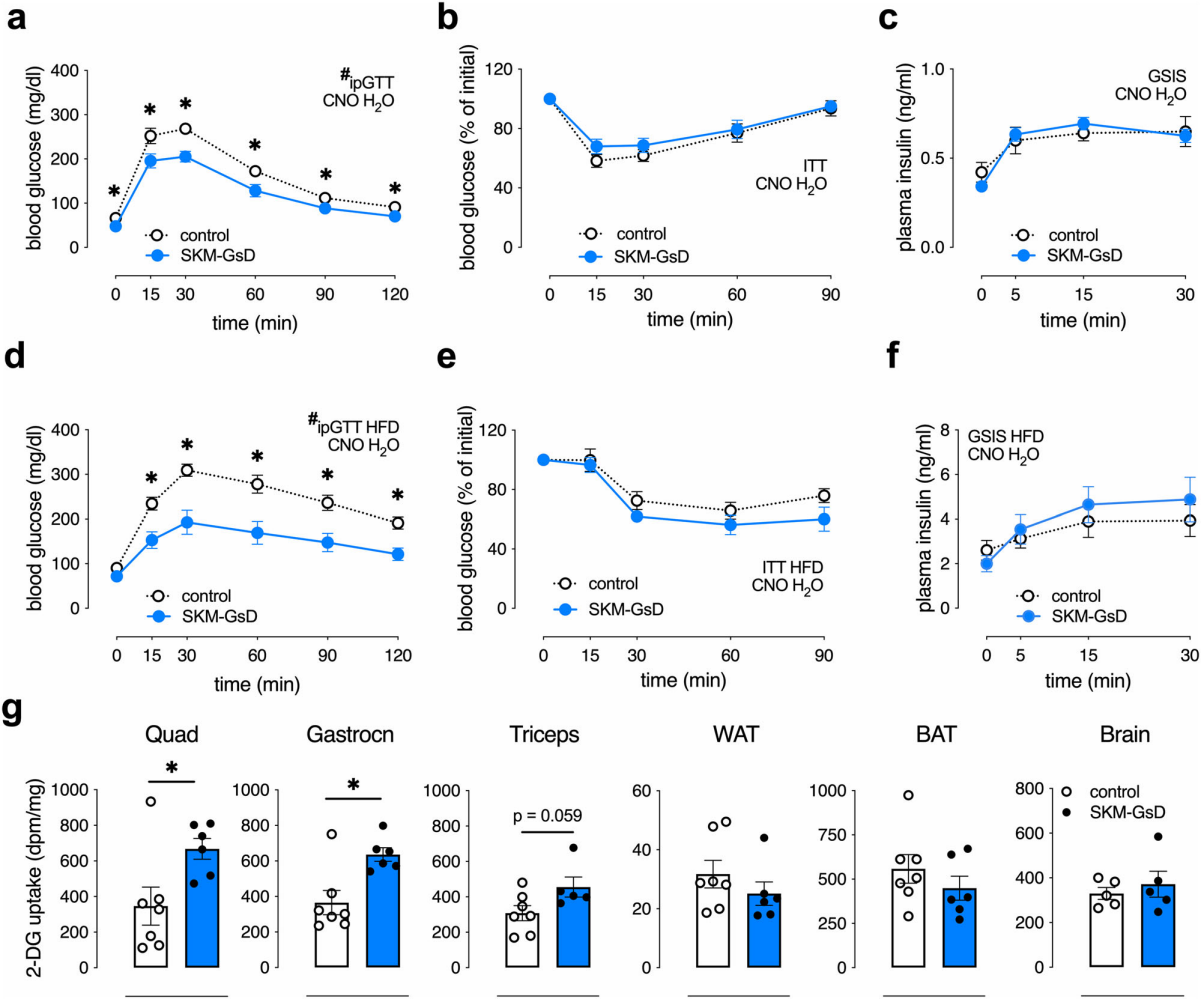
Chronic CNO treatment of SKM-Gs-DREADD mice improves glucose tolerance by enhancing glucose uptake into SKM. Prior to metabolic testing, control and SKM-GsD mice consumed drinking water containing CNO (250 mg/l; CNO H_2_O) for 7 days. All studies were carried out with male mice that were at least 8 weeks old. **a** Glucose tolerance test (GTT), lean control (*n* = 6), and SKM-GsD mice (*n* = 5, overnight fasted) injected i.p. with glucose (2 g/kg). *p < 0.05, Student’s *t*-test, #*p* < 0.05, repeated-measure two-way ANOVA, genotype effect. **b** Insulin tolerance test (ITT), lean control (*n* = 6) and SKM-GsD mice (*n* = 5, fasted for 4 h) injected i.p. with insulin (0.75 IU/kg). **c** Glucose-stimulated insulin secretion (GSIS), lean control (*n* = 6), and SKM-GsD mice (*n* = 5, overnight fasted) injected i.p. with glucose (2 g/kg). **d** GTT, HFD-induced obese control, and SKM-GsD mice (overnight fasted) injected i.p. with glucose (1 g/kg) (*n* = 7/group). *p < 0.05, Student’s *t*-test, #*p* < 0.05, repeated-measure two-way ANOVA, genotype effect. **e** ITT, HFD-induced obese control, and SKM-GsD mice (fasted for 4 h) injected i.p. with insulin (1.5 IU/kg) (*n* = 6/group). **f** GSIS, HFD-induced obese control (*n* = 7), and SKM-GsD mice (*n* = 6, overnight fasted) injected i.p. with glucose (1 g/kg). **g** In vivo 2-deoxyglucose (2-DG) uptake studies. Lean control (*n* = 7) and SKM-GsD mice (*n* = 6, overnight fasted) that were maintained on CNO drinking water for 7 days were injected with glucose (2 g/kg i.p.) containing 10 μCi of [^14^C]2-DG. Two hours after injections, tissues were collected and the [^14^C]2-DG-6-phosphate content was determined in the indicated tissues. Quad, quadriceps muscle; Gastrocn, gastrocnemius muscle; WAT, white adipose tissue; BAT, brown adipose tissue. Data are presented as means ± SEM. *p < 0.05 (unpaired two-tailed Student’s *t*-test). Source data are provided as a Source data file.

To further support the concept that activation of receptor-mediated G_s_ signaling in SKM causes pronounced metabolic improvements, control and SKM-GsD mice maintained on regular chow were treated with CNO (250 mg/l) via the drinking water for 1 week and then subjected to a series of metabolic tests. At the end of the CNO treatment period, male (Fig. 2a) and female (Supplementary Fig. 3g) SKM-GsD mice showed improved glucose tolerance and reduced fasting blood glucose levels (Supplementary Fig. 3h) without changes in body weight (Supplementary Fig. 3i), insulin tolerance (Fig. 2b), GSIS, and plasma insulin levels (Fig. 2c and Supplementary Fig. 3j). Importantly, CNO treatment also improved glucose homeostasis in HFD-induced obese mice (Fig. 2d and Supplementary Fig. 3l). As observed with lean SKM-GsD mice, CNO treatment of obese SKM-GsD mice had no significant effect on body weight (Supplementary Fig. 3k), insulin tolerance and GSIS (Fig. 2e, f, respectively). Thus, the metabolic phenotype observed with SKM-GsD mice treated with CNO closely mimicked the metabolic changes displayed by WT mice after clenbuterol treatment. Taken together, these findings strongly indicate that the metabolic improvements caused by clenbuterol treatment (Fig. 1) depend on the activation of G_s_ signaling in SKM (Fig. 2).

To investigate whether CNO treatment of SKM-GsD mice led to improved glucose uptake into SKM, we performed in vivo glucose uptake studies using [^14^C]-2-deoxyglucose (DG) as a tracer. We found that CNO-treated SKM-GsD mice showed a significant increase of glucose uptake into multiple SKM tissues (quadriceps and gastrocnemius muscles) (Fig. 2g). No changes in glucose uptake were observed in adipose tissues or the brain (Fig. 2g).

### Clenbuterol treatment increases glucose utilization in SKM

**Fig. 3:**
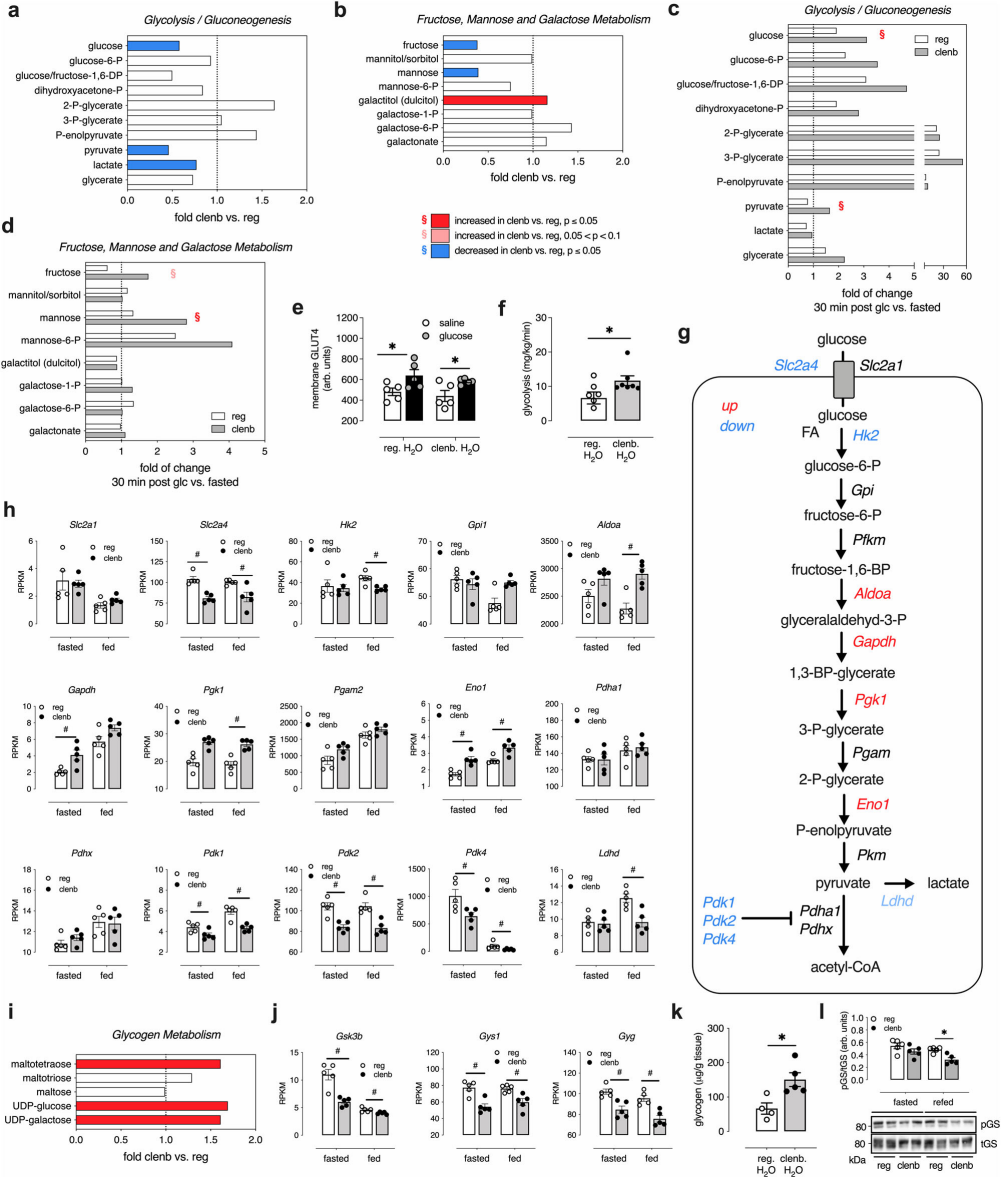
Chronic clenbuterol treatment of WT mice increases glucose utilization in SKM in vivo. Prior to metabolic testing, lean WT mice consumed drinking water containing clenbuterol (30 mg/l; clenb. H_2_O) or regular drinking water (reg. H_2_O) for 5 days. All studies were carried out with male mice that were ~12 weeks old. Quadriceps muscles were isolated for SKM analysis. **a**–**d**, **i** High-throughput metabolomic analysis was performed with SKM of WT mice treated with clenb. H_2_O or reg. H_2_O (*n* = 7/group). **a**, **b**, **i** Fold changes (clenb. vs. reg. H_2_O) in carbohydrate-related metabolites in SKM of fasted WT mice. **c**, **d** Fasted WT mice received an i.p. glucose bolus (2 g/kg) and SKM were isolated 30 min later. Bars represent fold changes in the indicated SKM metabolites 30 min after glucose (glc.) injection compared to the fasted state (30 min post glc. vs. fasted). As indicated in the insert below **b**, the color code represents a significant up- or downregulation (ANOVA contrast, clenbuterol effect) of metabolites (red or blue, respectively). ^§^Significant interaction effect between clenbuterol and glucose treatment (two-way ANOVA). **e** GLUT4 translocation studies. Lean WT mice were injected i.p. with glucose (2 g/kg) or saline and SKM were isolated for histological analysis of plasma membrane GLUT4 levels 15 min later (*n* = 5/group), *p < 0.05 for glucose effect (two-way ANOVA). **f** Glycolysis rate determined in plasma samples by measuring the rate of production of ^3^H_2_O from 3-^3^H-glucose (*n* = 7/group). **g** Graphic representation of differentially expressed genes within the glycolysis pathway in SKM of clenb. H_2_O vs. reg. H_2_O mice. **h** Changes of SKM transcripts encoding proteins involved in the glycolysis pathway. SKM gene expression levels (RPKM) in **h** and **j** were determined via RNA-Seq in fasted and freely fed WT mice. #Significantly regulated (two-tailed Wald test (DESeq2)) (*n* = 5/group, adjusted *p*-value < 0.05). **i** Fold changes (clenb. vs. reg. H_2_O) in glycogen metabolism-related metabolites in SKM of fasted WT mice (*n* = 7/group). **j** Changes of SKM transcripts coding for proteins involved in glycogen metabolism. **k** Glycogen levels of SKM of freely fed WT mice (*n* = 4 on reg. H_2_O, *n* = 5 on clenb. H_2_O) *p < 0.05 (unpaired two-tailed Student’s *t*-test). **l** SKM glycogen synthase (GS) phosphorylation (pGS). Following consumption of clenb. or reg. H_2_O, lean WT mice (*n* = 5/group) were fasted for 24 h and then given access to food for 6 h (refed) before SKM was collected for western blotting. Quantification and representative blots of GS phosphorylation. *p < 0.05 (two-way ANOVA followed by Sidak’s post hoc test). Data are presented as means ± SEM. RPKM, reads per kilobase of transcript per million of mapped reads. Source data are provided as a Source data file.

**Fig. 4:**
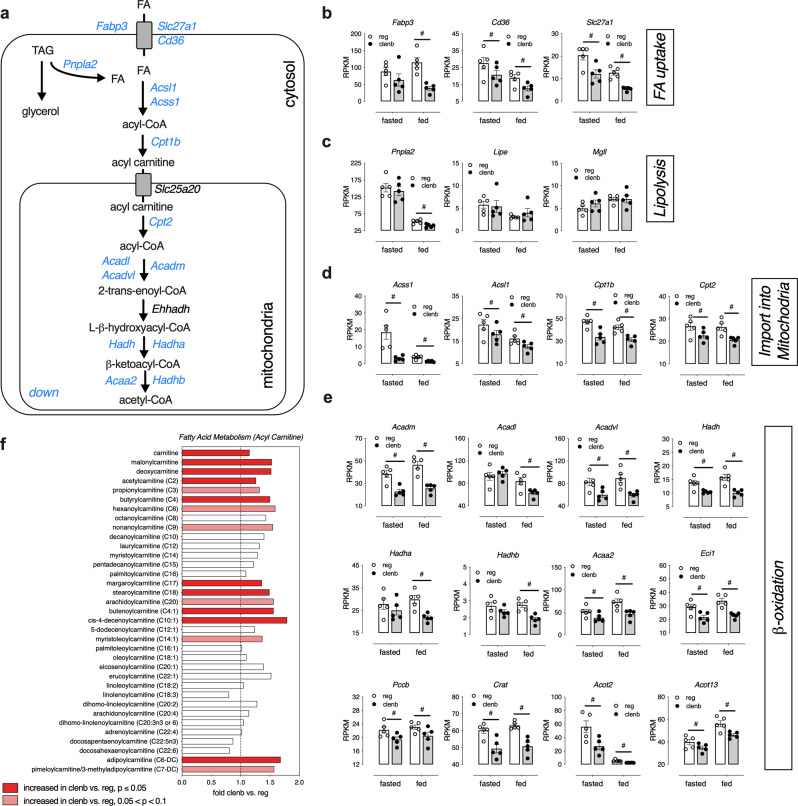
Chronic clenbuterol treatment decreases fatty acid oxidation in SKM in vivo. Prior to metabolomics and gene expression studies, lean WT mice consumed drinking water containing clenbuterol (30 mg/l; clenb. H_2_O) or regular drinking water (reg. H_2_O) for 5 days. All studies were carried out with male mice that were ~12 weeks old. **a** Graphic representation of differentially expressed genes within the fatty acid oxidation pathway in SKM (quadriceps muscle) of clenb. H_2_O vs. reg. H_2_O mice (*n* = 5/group). **b**–**e** Changes in the levels of SKM transcripts involved in fatty acid (FA) uptake (**b**), lipolysis (**c**), import of FAs into mitochondria (**d**), and FA β-oxidation (**e**). SKM gene expression levels (RPKM) were determined via RNA-Seq in fasted (15 h) and freely fed WT mice by using the Genomatix Genome Analyzer platform. Data are presented as means ± SEM. ^#^Significantly regulated (two-tailed Wald test (DESeq2)) (*n* = 5/group, adjusted *p*-value < 0.05). **f** Fold changes (clenb. vs. reg. H_2_O) of acylcarnitine levels in SKM of fasted (15 h) WT mice, determined by high-throughput metabolomics (*n* = 7/group). The red bars indicate significant clenbuterol effects (ANOVA contrast). RPKM, reads per kilobase of transcript per million of mapped reads. Source data are provided as a Source data file.

To explore the changes in SKM cell metabolism following long-term clenbuterol treatment, we used unbiased high-throughput metabolomics and transcriptomics approaches. Metabolomic analysis of SKM (quadriceps muscle) from lean WT mice treated with clenbuterol or regular water identified a total of 648 metabolites. Principal component analysis showed a clear separation of the two experimental groups based on their biochemical profile (Supplementary Fig. 4a). Clenbuterol treatment affected the SKM metabolites of all macronutrient classes (Supplementary Fig. 4b and Supplementary Data 1), leading to changes in the levels of glucose metabolites (Fig. 3a–d, i), lipids (Fig. 4f and Supplementary Fig. 5g), ketone bodies (Supplementary Fig. 5c), and amino acids (Supplementary Fig. 6a, b). In agreement with these extensive changes in metabolite levels, transcriptome analysis identified >4000 differentially expressed genes that are involved in multiple metabolically relevant pathways (Supplementary Fig. 4c).

Given the central role of glucose in SKM metabolism and the outcome of our in vivo metabolic studies, we next closely examined metabolites in glucose-related pathways. In the fasted state, WT mice that had been treated with clenbuterol for 5 days showed significantly lower SKM glucose, pyruvate, lactate, fructose, and mannose levels (Fig. 3a, b), as compared to untreated WT mice, suggesting that clenbuterol treatment promoted the use of these metabolites as fuel source. When control and clenbuterol-treated mice were injected with an i.p. glucose bolus, the clenbuterol group showed lower blood glucose levels (215 ± 15 vs. 272 ± 14 mg/dl) and a significantly greater increase in SKM levels of glucose and pyruvate (Fig. 3c) and sugars such as fructose and mannose (Fig. 3d) 30 min after glucose injection, in agreement with increased glucose transport into SKM.

Interestingly, SKM GLUT1 protein levels and glucose-induced SKM GLUT4 translocation to the plasma membrane were not significantly different between the two groups of mice (Fig. 3e and Supplementary Fig. S7a–c), indicating that the ability of clenbuterol to alter SKM metabolism and improve glucose tolerance is not due to enhanced GLUT1 (total) or GLUT4 levels in the plasma membrane. Thus, the most likely scenario is that the clenbuterol-stimulated increase in glucose metabolism drives glucose uptake into SKM.

To further test this hypothesis, we measured the glycolysis rate in mice that had been treated with clenbuterol. We injected fasted lean WT mice with glucose containing a 3-^3^H-glucose tracer, collected plasma samples at multiple time points following the glucose bolus, and then determined the rate of ^3^H_2_O production from 3-^3^H-glucose as a measure of glycolysis rate. Indeed, clenbuterol-treated mice showed a significantly higher glycolysis rate than control mice that had not received clenbuterol (Fig. 3f). These data strongly support the concept that clenbuterol leads to an increase in glucose utilization in SKM.

Moreover, SKM RNA-Seq data obtained from clenbuterol-treated WT mice showed an upregulation of several genes encoding glycolytic enzymes including *Aldoa*, *Gapdh*, *Pgk1*, and *Eno1* (Fig. 3g, h), as compared to SKM from control mice that had not received clenbuterol. Further, the expression of *Pdk1*, *Pdk2*, and *Pdk4*, the kinases that phosphorylate and inhibit pyruvate dehydrogenase, the rate-limiting enzyme of glucose oxidation, was significantly decreased in SKM of clenbuterol-treated mice (Fig. 3g, h).

In addition, metabolomic analysis showed that SKM of fasted clenbuterol-treated mice displayed higher levels of glycogen breakdown intermediates and precursors, including maltotetraose and UDP-glucose (Fig. 3i). The clenbuterol-induced changes in the SKM levels of glycogen intermediates were accompanied by significantly lower expression levels of genes involved in the regulation of glycogen metabolism including *Gsk3b*, *Gys1*, and *Gyg* (Fig. 3j). Importantly, WT mice that had free access to food and were treated with clenbuterol showed a significant increase in SKM glycogen levels, as compared to control littermates that had not received clenbuterol (Fig. 3k). Consistent with the increased glycogen synthesis, mice that had been fasted for 24 h and then refed for 6 h showed significantly lower levels of phosphorylated GS (S641) in SKM (Fig. 3l).

In summary, the above data clearly indicate that chronic clenbuterol treatment leads to a metabolic reprogramming of SKM towards enhanced glucose utilization.

### Clenbuterol treatment affects fatty acid oxidation in SKM

While glucose utilization was enhanced in SKM of WT mice treated with clenbuterol (Fig. 3), gene expression and metabolomics pathway analyses also suggested extensive changes in pathways involved in SKM fat oxidation (Fig. 4 and Supplementary Fig. 4b, c). Clenbuterol administration resulted in reduced SKM expression levels of genes coding for proteins involved in fatty acid uptake (*Fabp3*, *Slc27a1*, *Cd36*; Fig. 4b), lipolysis (*Pnplap2*; Fig. 4c), import of fatty acids into mitochondria (*Acss1*, *Acsl1*, *Cpt1b*, *Cpt2*; Fig. 4d), and β-oxidation (*Acadm*, *Acadl*, *Acadvl*, *Hadh*, *Hadha*, *Hadhb*, *Acaa2*, *Eci1*, *Pccb*, *Crat*, *Acot2*, *Acot13*; Fig. 4e). Metabolomic analysis indicated that clenbuterol-treated mice showed increased levels of SKM acylcarnitines (e.g., malonylcarnitine), as compared to control animals that had not received clenbuterol (Fig. 4f). Following clenbuterol treatment, we also observed changes in the levels of several classes of complex lipids including glycerophospholipids and ceramides, and in metabolites linked to phospholipid synthesis and turnover including phosphoethanolamine and glycerophosphocholine (Supplementary Data 1).

We further characterized clenbuterol-induced changes in SKM fatty acid metabolism using labeled palmitate metabolites. To directly measure fatty acid uptake into SKM, we injected mice with [9,10-^3^H]-(R)-2-bromopalmitate ([^3^H]-BROMO), a radioactive labeled analog of palmitate that is not a substrate for β-oxidation, but becomes trapped in slower metabolic processes of ω- and α-oxidation^37^. We did not observe differences in [^3^H]-BROMO levels in SKM, liver, or adipose tissues of mice treated with clenbuterol water, as compared with mice consuming regular drinking water (Supplementary Fig. 8a). To determine whether the rate of β-oxidation was reduced in SKM of clenbuterol-treated mice, we injected overnight fasted mice with the uniformly labeled ^13^C stable isotope of palmitate ([U-^13^C]-palmitate, 50 μg/kg i.p.). We found that ^13^C enrichment in C_16_-, C_14_-, and C_12_-acylcarnitines was stable at 25 min after [U-^13^C]-palmitate injection, and that similar enrichment levels were achieved in SKM of control mice, indicative of a pseudo isotopic steady state (Supplementary Fig. 8b, c). Interestingly, although there were no changes in ^13^C enrichment in SKM palmitate (Supplementary Fig. 8b), we detected significantly higher levels of ^13^C-labeled C_14_-acylcarnitine in SKM of clenbuterol-treated mice (Supplementary Fig. 8c). No such changes were observed in the liver (Supplementary Fig. 8d, e). Since several genes within the β-oxidation pathway were downregulated in SKM following clenbuterol treatment (Fig. 4a, d, e), this observation supports the concept that clenbuterol treatment leads to a reduced rate of fatty acid oxidation in SKM.

To obtain additional information on the energy metabolism of mice maintained on clenbuterol drinking water, we subjected lean WT mice to indirect calorimetry measurements. After the recording of baseline parameters, mice were put on either clenbuterol or regular (vehicle) drinking water for 5 days (Supplementary Fig. 9a). As expected, after 5 days of clenbuterol treatment, mice showed significantly lower blood glucose levels (Supplementary Fig. 9b), as compared to mice maintained on regular drinking water. Clenbuterol administration did not cause any changes in food intake (Supplementary Fig. 9c) or locomotor activity (Supplementary Fig. 9d). However, we observed a slight increase in oxygen consumption (Supplementary Fig. 9e) and total energy expenditure (TEE; Supplementary Fig. 9f) in the clenbuterol-treated mice. More importantly, mice that received clenbuterol showed a significant increase in respiratory exchange ratio (RER; Supplementary Fig. 9g, h, j), specifically during the day/light period (Supplementary Fig. 9h) when mice are inactive. These results further corroborate that chronic clenbuterol treatment triggers a shift in SKM of preferential energy substrates from lipids to glucose, in agreement with the transcriptomic, metabolomic, and isotope labeling data (see above).

### Improvements in glucose homeostasis following clenbuterol treatment are dependent on mTORC2 and AMPK signaling

To further investigate the cellular mechanisms underlying the beneficial metabolic effects of clenbuterol treatment, we performed western blotting studies based on the top regulated signaling pathways (Supplementary Fig. 4c), focusing on selected protein targets known to be involved in the regulation of glucose homeostasis in SKM. Lean WT mice that had been treated with vehicle or clenbuterol via the drinking water for 5 days were fasted overnight and then injected with saline or glucose. Quadriceps muscles were isolated 30 min later and processed for immunoblotting experiments. As previously observed in the refeeding experiment (Fig. 3l), clenbuterol treatment had a significant effect on the phosphorylation status of SKM glycogen synthase (GS) and the upstream kinase, GSK3β (Supplementary Fig. 10a–c). While pGS (S641) was significantly increased in saline-injected clenbuterol mice (fasting conditions), glucose treatment led to a significant (activating) dephosphorylation of GS in clenbuterol-treated mice only (Supplementary Fig. 10b). These data suggest that the observed improvements in glucose metabolism are potentially linked to enhanced glycogen metabolism, showing increases in glycogen breakdown during fasting (Fig. 3i) and enhanced glycogen storage upon glucose availability (Fig. 3k).

We also found that clenbuterol treatment decreased the phosphorylation of AKT (Supplementary Fig. 10d–f) and mammalian target of rapamycin (mTOR) (S2481, Supplementary Fig. 10i) in SKM tissue from WT mice. However, these changes were mostly driven by increased protein expression of total AKT (Supplementary Fig. 10h) and mTOR (Supplementary Fig. 10k), and not the amount of phosphorylated proteins per se (Supplementary Fig. 10g, j). Total AMPK was also slightly increased in SKM of clenbuterol-treated mice (Supplementary Fig. 10n). Interestingly, while a glucose bolus reduced AMPK phosphorylation in control animals (Supplementary Fig. 10a, l, m), pAMPK was already reduced in the clenbuterol group prior to glucose administration, and glucose treatment did not lead to a further reduction of the low pAMPK levels (Supplementary Fig. 10a, l, m).

**Fig. 5:**
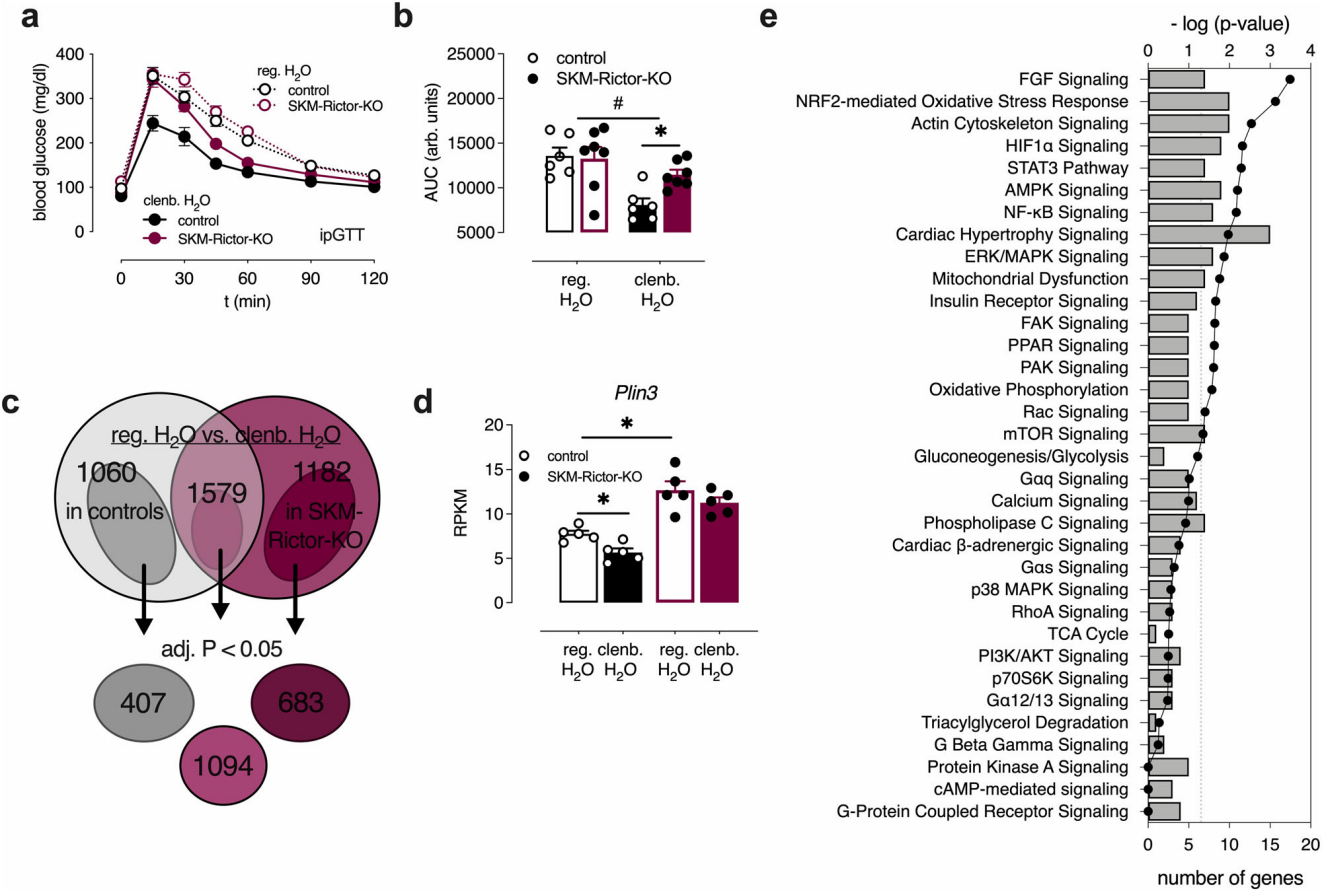
Chronic clenbuterol treatment causes beneficial metabolic effects through SKM mTORC2 signaling. **a** Glucose tolerance test (GTT) with male lean control (*n* = 6) and SKM-Rictor-KO mice (*n* = 7, overnight fasted) after consumption of regular drinking water (reg. H_2_O) or clenbuterol-containing drinking water (clenb. H_2_O) for 5 days. **b** Comparison of Area under the curve (AUC) values for the data shown in **a**. #*p* < 0.05 for clenbuterol effect (two-way ANOVA); *p < 0.05 (two-way ANOVA, followed by Sidak’s post hoc test), *p* = 0.06 for clenbuterol × genotype interaction effect (two-way ANOVA). **c** Intersection of differentially expressed genes in SKM (gastrocnemius muscle) of control vs. SKM-Rictor-KO mice, either maintained on clenbuterol or regular drinking water (*n* = 5/group). Adjusted *p*-value of <0.1 was used for the initial analysis to filter out genes that have narrowly missed statistical significance of the standard adjusted *p*-value < 0.05. After identifying the differentially expressed genes that were common to both groups (1579 genes) or different in control mice only (1060 genes) or SKM-Rictor-KO only (1182 genes), an adjusted *p*-value cutoff of 0.05 was applied to these 3 groups. **d** *Plin3* expression levels determined by RNA-Seq analysis. *Significantly regulated by two-tailed Wald’s test (DESeq2) (*n* = 5/group, adjusted *p*-value < 0.05). **e** Enrichment of signaling pathways of differentially expressed genes in SKM of only control mice maintained on clenbuterol vs. regular drinking water (407 genes from **c**), determined via Ingenuity Pathway Analyzer (Qiagen). Data in **a**, **b**, and **d** are presented as means ± SEM. Source data are provided as a Source data file.

Pathway analyses of differentially expressed genes following clenbuterol treatment (Supplementary Fig. 4c) showed enrichment of genes regulated by mTOR and AMPK. Indeed, previous in vitro studies demonstrated that isoproterenol, a non-subtype-selective β-AR agonist, could promote glucose uptake in the L6 muscle cell line in an mTORC2-dependent manner^18^, raising the possibility that mTORC2 may play a role in mediating the beneficial metabolic effects of SKM β_2_-AR stimulation. To test this hypothesis, we carried out metabolic studies with mutant mice harboring an SKM-specific deletion of Rictor, a critical subunit of mTORC2 (SKM-Rictor-KO mice). We found that the improvements in glucose homeostasis caused by clenbuterol treatment were significantly reduced in SKM-Rictor-KO mice (Fig. 5a, b). On the other hand, treatment of WT mice with rapamycin (Supplementary Fig. 10o), for 24 h via the drinking water, 10 mg/l), an inhibitor of mTORC1, did not affect the improvement in glucose tolerance caused by clenbuterol treatment (Supplementary Fig. 10p, q). Moreover, clenbuterol treatment failed to increase SKM glycogen levels in the absence of SKM mTORC2 (Supplementary Fig. 10r).

The importance of SKM mTORC2 signaling in mediating the β_2_-AR-dependent beneficial metabolic effects was further examined by transcriptome analysis of clenbuterol-treated SKM-Rictor-KO mice, as compared to clenbuterol-treated control littermates. To identify genes that were regulated by clenbuterol in control mice, but not in SKM-Rictor-KO mice, a less stringent cut-off for the adjusted *p*-value (<0.1) was set for the initial analysis. SKM (gastrocnemius muscle) samples derived from control mice treated with clenbuterol showed 2639 differentially expressed genes (1336 upregulated, 1303 downregulated, adjusted *p* < 0.1), as compared to vehicle-treated mice (Fig. 5c). SKM samples from clenbuterol-treated SKM-Rictor-KO mice showed a similar number (2761) of differentially expressed genes (1398 upregulated, 1363 downregulated, adjusted *p* < 0.1), as compared to SKM samples from vehicle-treated SKM-Rictor-KO mice (Fig. 5c). Interestingly, there was an overlap of 1579 differentially expressed genes in these two data sets (Fig. 5c). Somewhat surprisingly, the clenbuterol-induced changes in gene expression of the major metabolite pathways (Supplementary Table 1) did not require the presence of SKM mTORC2, as clenbuterol caused similar alterations in gene expression profiles of SKM-Rictor-KO and control mice (Supplementary Table 1). However, we also identified many genes (407 and 683 (adjusted *p* < 0.05), respectively) that were differentially expressed only in SKM tissue of clenbuterol-treated control mice or clenbuterol-treated SKM-Rictor-KO mice, respectively (Fig. 5c). Among these genes, the perilipin 3 gene (*Plin3)* caught our particular attention, because *Plin3* represents a regulator of lipid metabolism downstream of the mTORC2 complex^38^. Previous work has shown that SKM-Rictor-KO mice shift to lipids as energy source by increasing Plin3 expression^38^. Clenbuterol treatment significantly decreased *Plin3* expression in SKM of control mice, but not in SKM-Rictor-KO mice, where SKM *Plin3* expression was increased in the absence or presence of clenbuterol (Fig. 5d), providing a possible link between clenbuterol and mTORC2 in regulation of energy sources.

AMPK and mTORC2-dependent signaling pathways differentially regulate *Plin3* expression^38^. As the AMPK signaling pathway was among the significantly enriched pathways in SKM of clenbuterol-treated mice (Fig. 5e and Supplementary Fig. 4c) and SKM levels of endogenous AICAR and other adenosine derivatives were elevated (Supplementary Fig. 10s), we next investigated the potential role of SKM AMPK in mediating the beneficial metabolic effects of clenbuterol administration. For these experiments, we used transgenic mice that expressed a dominant-negative version of AMPK under the transcriptional control of the muscle-specific MCK promoter (MCK-AMPK-DN)^39^. Remarkably, as observed with the SKM-Rictor-KO mice (Fig. 5a, b), the ability of clenbuterol treatment to improve glucose tolerance was nearly abolished in the MCK-AMPK-DN mice (Supplementary Fig. 10t, u).

In sum, these data clearly indicate that chronic clenbuterol treatment regulates SKM signaling via multiple metabolic sensors and signaling proteins, including β_2_-AR, G_s_, mTORC2, and AMPK.

## Discussion

The present study has led to several important findings regarding the cellular and molecular mechanisms by which chronic clenbuterol treatment improves glucose homeostasis and other metabolic functions. We provide convincing evidence that the beneficial metabolic effects of chronic clenbuterol depend on the stimulation of SKM β_2_-ARs, and that the coupling of these receptors to SKM G_s_ is essential for the manifestation of the clenbuterol-induced metabolic improvements. These conclusions are supported by studies using a newly developed mouse model (SKM-GsD mice) that allowed us to monitor the metabolic outcome of selective activation of SKM G_s_ signaling. In addition, comprehensive unbiased metabolomic and RNA-Seq studies provided important clues regarding the molecular mechanisms underlying the clenbuterol-induced improvements in SKM glucose homeostasis.

Specifically, we found that chronic clenbuterol treatment significantly enhanced glucose tolerance in lean and obese WT mice and the STZ model of β-cell dysfunction, without increasing plasma insulin levels or insulin tolerance (Fig. 1a–e). This effect was absent in SKM β_2_-AR-KO and SKM-Gs-KO mice (Fig. 1g, h), indicating that the beneficial metabolic effects of systemic, chronic administration of the selective β_2_-AR agonist are mediated by SKM β_2_-ARs signaling via G_s_. We previously showed that SKM-β-arrestins do not contribute to the beneficial metabolic effects caused by clenbuterol administration^19^. Although our study specifically focused on SKM, our findings also suggest that previously reported changes in mouse liver metabolism following prolonged treatment with clenbuterol^40^ may be secondary to the action of clenbuterol at SKM β_2_-AR.

The SKM-specific expression of a CNO-sensitive, GsD^32–36^ allowed us to study the in vivo metabolic consequences of selectively activating G_s_ in SKM in vivo. CNO treatment of SKM-GsD mice led to improved glucose tolerance (Fig. 2a, d and Supplementary Fig. S3g) by increasing glucose uptake into SKM in vivo (Fig. 2g). These findings mirror the effects observed after clenbuterol-induced activation of β_2_-ARs in WT mice (Fig. 1). Taken together, these data further corroborate the central role of SKM β_2_-AR/Gα_s_ signaling in meditating the metabolic effects caused by systemic administration of a β_2_-AR agonist.

Chen et al.^41^ previously demonstrated that mice lacking Gα_s_ selectively in SKM display decreased glucose tolerance with no changes in insulin secretion or sensitivity. These data support our finding that activation of the β_2_-AR/Gα_s_ pathway in SKM improves glucose homeostasis in an insulin-independent manner (Figs. 1b and 2b).

Previous work by the Lefkowitz lab has shown that both the β_1_- and β_2_-ARs can undergo PKA-dependent “G_s_ to G_i_ switching”^12,42^. On the basis of these studies, we cannot completely rule out the possibility of a role for sequential signaling via G_s_ and G_i_.

Surprisingly, the usual suspects in mediating improved glucose tolerance and glucose uptake in SKM, GLUT1, and GLUT4 did not show increased expression levels or enhanced glucose-induced translocation to the plasma membrane following clenbuterol treatment (Fig. 3e and Supplementary Fig. S7a–c). In contrast, previous in vitro studies suggested that β_2_-AR stimulation leads to enhanced GLUT4 translocation in cultured muscle cells^18,20^. These discrepant findings are most likely due to differences in experimental conditions. While the in vitro studies were performed with cultured cell lines overexpressing an epitope-tagged GLUT4 construct and short incubations with clenbuterol, the present study was carried out with isolated SKM prepared from WT mice treated with clenbuterol in vivo for a prolonged period of time and examined the translocation of endogenous GLUT4 following a glucose bolus.

Using unbiased transcriptomic and metabolomic approaches (Fig. 3 and Supplementary Fig. S4), we showed that clenbuterol treatment of WT mice caused a metabolic reprogramming, leading to enhanced glucose utilization in SKM. Although clenbuterol did not cause any changes in fat mass (Supplementary Fig. 2c), the expression levels of several key genes involved in fatty acid metabolism and oxidation, including carnitine palmitoyltransferase-1b (*Cpt1b*), were significantly downregulated in SKM following treatment with clenbuterol (Fig. 4a–e and Supplementary Table 1). These changes were accompanied by increased levels of acylcarnitines in SKM (Fig. 4f), further supporting the concept that chronic clenbuterol impairs fatty acid oxidation in SKM. This conclusion is also supported by the outcome of an experiment using a stable palmitate isotope (Supplementary Fig. 8b, c), as suggested by higher levels of C_14_-acylcarnitines.

Decreased SKM oxidative capacity is often associated with insulin resistance^2^. However, clenbuterol treatment did not affect insulin tolerance, but improved glucose tolerance with reduced insulin levels (Fig. 1a–e). The analysis of gene expression and functional data indicated that clenbuterol also increased the rate of glycolysis in SKM (Fig. 3a–c, f–h and Supplementary Fig. S9h), indicative of a shift in fuel utilization from fatty acid oxidation to increased glucose utilization to sustain SKM energy supply. Interestingly, mice lacking *Cpt1b* specifically in SKM show similar changes in glucose homeostasis as seen after clenbuterol treatment of WT mice, including impaired fatty acid oxidation^43^, increased malonyl- and succinyl-carnitine levels, improved glucose tolerance, and enhanced basal glucose uptake in primary myotubes that is insulin-independent, indicative of a compensatory adaptation towards enhanced glucose usage^44^. Moreover, the metabolic changes displayed by the SKM *Cpt1b* KO mice were dependent on increased signaling through the AMPK and mTORC2 pathways^44^. In line with this observation, we demonstrated that inhibition of either of these two pathways interfered with the ability of clenbuterol to improve glucose tolerance (Fig. 5a, b and Supplementary Fig. S10t–u). In agreement with our findings, independent activation of either AMPK^45^ or mTORC2^46^ has been shown to increase SKM glycolysis rate. Furthermore, G_s_ signaling has been shown to activate AMPK under certain circumstances^47^, which can interact with mTORC2 during energetic stress^48,49^. These findings provide a possible link between enhanced SKM β_2_-AR/G_s_ signaling and the increases in SKM AMPK and mTORC2 activity observed in the present study.

Somewhat surprisingly, the clenbuterol-induced changes in the expression of genes involved in major metabolic pathways did not require the presence of SKM mTORC2, as clenbuterol caused similar alterations in gene expression profiles in SKM-Rictor-KO and control mice (Supplementary Table 1). This observation suggests that SKM mTORC2 modifies SKM cellular functions primarily at the posttranscriptional level (following clenbuterol treatment) or in another manner that does not require changes at the transcriptional level. Thus, the precise mechanisms through which mTORC2 contributes to the beneficial metabolic effect of SKM β_2_-AR stimulation requires more detailed future investigation.

Impaired glycogen synthesis in SKM is considered the earliest metabolic defect in the development of T2D^2^. Interestingly, chronic clenbuterol treatment enhanced glycogen storage in SKM of freely fed mice (Fig. 3k), whereas fasted mice displayed increased levels of glycogen breakdown intermediates such as maltotetraose^50^ and UDP-glucose (Fig. 3i), indicating that glycogen represents an important source of glucose as energy substrate under fasting conditions. We found that SKM β_2_-AR stimulation regulated SKM glycogen metabolism both at the transcriptional and posttranslational level (through protein phosphorylation). Clenbuterol treatment decreased SKM gene expression levels of glycogenin (*Gyg*) and *Gsk3b* (Fig. 3j), two key regulators of glycogen synthesis. In agreement with these data, Gyg*-*deficient mice also showed increased glycogen levels in SKM and a switch to a more glycolysis-based fuel usage in SKM^51^. Similarly, SKM-specific GSK3β-KO mice display improved glucose tolerance and enhanced SKM glycogen deposition^52^. Moreover, consistent with our observation that clenbuterol treatment increased endogenous AICAR levels (Supplementary Fig. 10s) and required SKM AMPK to mediate its beneficial effects (Supplementary Fig. 10t–u), long-term activation of AMPK with AICAR led to increased glycogen accumulation in mouse or rat SKM^45,53^.

Long-term treatment of human subjects with a β_2_-AR agonist (terbutaline) led to an increase in non-oxidative disposal of glucose, e.g., in form of glycogen; however, the cellular mechanisms underlying this effect were not explored^54^.

In the present study, clenbuterol treatment significantly affected the inhibitory phosphorylation of GS (pGS) (Supplementary Fig. 10a, b), based on glucose availability. In the fasted state (12 h, low blood glucose levels), clenbuterol administration increased SKM pGS levels (Supplementary Fig. 10b), consistent with a shift towards glycogen breakdown (Fig. 3i). In contrast, following glucose injection, SKM pGS levels significantly declined in clenbuterol-treated mice only (Supplementary Fig. 10a, b). A similar regulation of SKM pGS was observed when mice were refed after a 24 h-fasting period (Fig. 3l), indicative of the key role of SKM β_2_-ARs in regulating SKM glycogen metabolism in a glucose/fuel-dependent manner. More detailed studies involving the infusion of stable glucose isotopes will be required to clearly define the fractions of glucose cycled via the glycogen and glycolysis pathways in SKM following clenbuterol treatment.

Although clenbuterol-mediated SKM hypertrophy was not the focus of this study, it is worth mentioning that the 5-day clenbuterol treatment led to a significant increase of several amino acids in SKM, following administration of a glucose bolus (Supplementary Fig. 6b). One possible explanation for this observation is that those mostly glucogenic amino acids are conserved for SKM protein synthesis in the presence of available glucose as a fuel source. However, the non-essential amino acids might also have been synthesized from glycolysis intermediates (Supplementary Fig. 6c, d). In agreement with this observation, it is known that glucose is rapidly converted into amino acids and other compounds/metabolites in SKM^55^, and that glucose infusion significantly increases protein synthesis^56^. As the clenbuterol-induced shift in preferential substrate utilization occurred in the absence of a significant increase in lean mass (Supplementary Fig. 2b), it is possible that the clenbuterol-mediated reprogramming of SKM towards enhanced glucose utilization also drives SKM hypertrophy that develops after clenbuterol treatment for several weeks^16^. Moreover, when fatty acid oxidation is impaired, amino acids can represent an important fuel source^43^. Higher levels of *N*-acetylated amino acids (Supplementary Data 1), propionyl-carnitine (Fig. 4f), and succinyl-carnitine (Supplementary Fig. 5e) in clenbuterol-treated mice are potential indicators of increased amino acid catabolism/oxidation under fasting conditions.

A previous histological study reported that long-term β_2_-AR stimulation promotes a slow-to-fast muscle fiber switch (oxidative-to-glycolytic)^57^. Although our SKM RNA-Seq data did not reveal changes in classic muscle fiber markers like Myh7, Myh1, and Myh2, our unbiased metabolomic and transcriptomic approaches provided evidence for β_2_-AR-mediated SKM metabolic reprogramming towards enhanced glucose utilization at both the transcriptional and metabolite level, consistent with the outcome of the study be Zeman et al.^57^.

The β_2_-AR is also abundantly expressed in human SKM (https://www.genecards.org/cgi-bin/carddisp.pl?gene=ADRB2), suggesting that clenbuterol may also cause beneficial effects on glucose homeostasis in humans. Clenbuterol and other β_2_-AR agonists are well known for their therapeutic potential in treating muscle wasting and related SKM disorders^16^. Moreover, clenbuterol is used for the treatment of asthma and related pulmonary diseases in several European and Asian countries (http://drugapprovalsint.com/clenbuterol/), due to its ability to act on bronchial smooth muscle β_2_-ARs. Kim et al.^58^ recently reported that clenbuterol-induced SKM hypertrophy in mice requires SKM β-arrestin-1 signaling. As the beneficial effects of clenbuterol on glucose homeostasis are mediated by SKM G_s_ signaling, it should be possible to develop novel classes of β_2_-AR agonists that stimulate SKM G_s_ but do not recruit β-arrestins. The data by Kim et al.^58^ suggest that the potential use of such G protein-biased β_2_-AR agonists for the treatment of SKM insulin resistance will not lead to the development of SKM hypertrophy.

In conclusion, by analyzing multiple new mouse models and using unbiased multiomic approaches, we provide clear evidence that systemic administration of a selective β_2_-AR agonist (clenbuterol) significantly improves glucose homeostasis under physiological and pathophysiological conditions. We demonstrated that the metabolic improvements observed after a 5-day clenbuterol administration require the activation of the SKM β_2_-AR/G_s_ complex and downstream AMPK and mTORC2 signaling. These signaling events eventually lead to a metabolic reprogramming of SKM towards enhanced glucose utilization. No other tissues or G protein signaling pathways appear to be involved in the clenbuterol-dependent metabolic improvements.

Our data strongly suggest that activation of G_s_ signaling in SKM (e.g., by the activation of G_s_-coupled receptors endogenously expressed by SKM) or of components of downstream signaling pathways represents an attractive approach to develop novel classes of antidiabetic drugs targeted at modifying SKM metabolism.

### Study limitations

Our study primarily focused on SKM phenotypes and did not explore the effect of systemic clenbuterol on other tissues expressing β_2_-ARs. Although we clearly demonstrated that the beneficial metabolic effects seen after a 5-day clenbuterol treatment are dependent on SKM-β_2_-ARs, we cannot completely exclude the possibility that the clenbuterol-induced changes in SKM (e.g., changes in gene transcription and metabolite levels) cause secondary changes in other tissues. The static metabolomic analyses that we performed provide a good overview of changes in SKM metabolite levels; however, infusion studies with isotope-labeled metabolite tracers are needed to draw clear conclusions about metabolite fluxes. Moreover, the precise molecular mechanisms through which chronic activation of SKM β_2_-AR affects AMPK and mTORC2 signaling to alter SKM fuel utilization remain to be explored in future studies.

## Methods

### Materials

The sources of antibodies, chemicals, assay kits, and mouse lines used in this study are listed in Supplementary Table 2.

### Mouse maintenance and diet

All mice were fed ad libitum and kept on a 12 h light, 12 h dark cycle at room temperature. Mice were maintained either on a standard mouse chow (7022 NIH-07 diet, 15% kcal fat, energy density 3.1 kcal/g, Envigo, Inc.) or a HFD (60% kcal from fat, energy density 5.49 kcal/g; # F3282, Bioserv) for at least 8 weeks.

All animal studies were carried out according to the US National Institutes of Health Guidelines for Animal Research and were approved by the NIDDK Institutional Animal Care and Use Committee.

### Generation of SKM-specific KO and knock-in mice

To generate SKM-specific KO and knock-in mice, floxed mice (see Supplementary Table 2 for specific floxed lines) were crossed with HSA-Cre(ER^T2^) mice^59^. To avoid potential developmental changes, Cre recombinase activity was induced in 7- to 8-week-old HSA-Cre(ER^T2^)-positive floxed/floxed mice by i.p. injection with tamoxifen (2 mg per day dissolved in corn oil) for 5 consecutive days. Tamoxifen-injected Cre-negative floxed/floxed littermates served as control mice.

### In vivo metabolic studies

Unless stated otherwise, all metabolic tests were performed on male littermates (age range: 10–30 weeks) using standard protocols. For i.p. GTTs (ipGTT), mice were fasted overnight for 15 h and blood glucose levels were measured before (0 min) and at defined time points after i.p. injection of normal saline containing glucose (2 g/kg for mice consuming normal chow; 1 g/kg for mice on HFD). For ITTs, mice were fasted for 4 h and blood glucose levels were measured before (0 min) and at indicated time points after i.p. injection of human insulin (0.75 IU/kg or 1 IU/kg, as indicated; Humulin R, Eli Lilly). Blood glucose levels were determined using a manual blood glucose meter (Contour; Bayer). To study GSIS, mice were fasted overnight for 15 h and blood samples were collected in heparinized capillary tubes (Fisher Scientific) before (0 min) and 5, 15, and 30 min following the i.p. injection of glucose (2 g/kg or 1 g/kg, as indicated). Samples were centrifuged (5000 × *g*, 10 min, 4 °C) for plasma collection and plasma insulin levels were determined using an ultra-sensitive mouse insulin ELISA kit (Crystal Chem, Inc.).

### Clenbuterol administration

For chronic clenbuterol treatment studies, WT mice consumed drinking water containing clenbuterol (30 mg/l) or regular drinking water for 5 days prior to metabolic experiments or tissue collection. During this 5-day period, mice consumed 3.5 ± 0.06 ml of clenbuterol water per day, resulting in an average clenbuterol dose of 104.7 ± 1.2 μg per mouse per day. For chronic clenbuterol studies performed with transgenic mice, baseline ipGTTs were performed with control and the indicated KO mice as described above. Two days later, both control and mutant mice started to consume clenbuterol drinking water. After 5 days on clenbuterol water, mice were subjected to another ipGTT.

### CNO administration

For long-term stimulation of G_s_ signaling in SKM, control and SKM-GsD mice received CNO continuously for 7 days by mixing CNO into the drinking water at a concentration of 250 mg/l, as described previously^8^.

### STZ model

To induce β-cell dysfunction, STZ treatment protocol has been used^28^, which has been previously shown to destroy about 70–80% of β-cells. Specifically, lean WT mice were fasted for 5 h and injected with a relatively low dose (50 mg/kg i.p.) of STZ for 5 consecutive days. Metabolic studies were initiated seven days after the last STZ injection.

### Rapamycin administration

To investigate whether activation of the mTORC1 complex contributes to the metabolic effects of long-term clenbuterol treatment, rapamycin (10 mg/l) or vehicle was provided for the initial 24 h of a 36 h treatment with clenbuterol drinking water. These experimental conditions were chosen to selectively inhibit the mTORC1 complex^60,61^ (see Supplementary Fig. 10o for a scheme of the experimental design).

### Body composition analysis

Mouse body mass composition (lean and fat mass) was determined using a 3-in-1 EchoMRI Analyzer (Echo Medical System).

### Indirect calorimetry measurements

TEE, RER (O_2_ consumed/CO_2_ produced), food intake, and locomotor activity (assayed by beam breaks) were measured simultaneously in mice housed at 22 °C using an Oxymax/CLAMS (Columbus Instruments). Sampling was performed every 13 min, measuring from 12 chambers. Mice were adapted in the chambers for 2 days and then baseline data were collected for 3 days. Water and food were provided ad libitum during the sampling period. For chronic clenbuterol studies, mice consumed clenbuterol (30 mg/l) or regular drinking water for 5 days. Treatment data were recorded for the last 3 days of clenbuterol treatment (see Supplementary Fig. 9a for a scheme of the experimental design). Data are presented as average parameters per day for the recorded period.

### In vivo [^14^C]2-deoxyglucose uptake

To measure glucose uptake into individual tissues in vivo, mice were fasted overnight for 14–16 h and then injected i.p. with saline containing 2 g/kg glucose and 10 μCi of [^14^C]2-deoxyglucose ([^14^C]2-DG) (Perkin Elmer). Mice were killed 2 h later, and SKM and other tissues were collected. The tissue content of [^14^C]2-DG-6-phosphate was determined as a measure of SKM glucose uptake, as described previously^62^.

### Glycolysis rate determination

Overnight (15 h) fasted mice were injected with glucose (2 g/kg i.p.) containing 2.5 mCi/kg [3-^3^H]-glucose. Blood samples were collected from the tail vein before and 5, 10, 15, 30, 45, and 60 min post injection. Plasma samples were processed as described previously and glycolysis rate was estimated as the rate of ^3^H_2_O appearance^63^.

### Glycogen measurements

Glycogen content in SKM was determined using a glycogen assay kit (Cayman Chemical), according to the manufacturer’s instructions.

### In vivo GLUT4 translocation

WT mice consumed clenbuterol (30 mg/l) or regular drinking water for 5 days. Mice were then fasted overnight (15 h) and injected i.p. with glucose (2 g/kg). Quadriceps muscles were isolated 15 min after the glucose bolus, mounted in Tissue-Tek®  medium, frozen in liquid nitrogen-cooled isopentane, and cryo-sectioned (7 μm) using a Leica CM3050S cryostat (chamber and object temperature was set to −22 °C). Sections were attached to glass slides and fixed for 10 min in ice-cold 4% paraformaldehyde in 0.1 M Sorensen’s phosphate buffer (pH 7.3), washed 3 × 10 min with 1× phosphate-buffered saline (PBS), and then incubated with a rabbit polyclonal antibody against GLUT4 (Thermo Scientific, PA5-23052; antibody was diluted in 1× PBS to 10 μg/ml; overnight incubation at 4 °C). Sections were washed 3 × 10 min and then incubated for 1 h with a goat anti-rabbit F(ab’)_2_ fragment-specific antibody conjugated to Alexa-594 (Jackson ImmunoResearch, 111-585-047; antibody dilution: 3.75 μg/ml in 1× PBS). Sections were washed 3 × 10 min in 1× PBS and incubated for 15 min with wheat germ agglutinin (WGA) conjugated to Alexa-488 diluted to 5 μg/ml in 1× PBS, followed by staining for 5 min with Hoechst 33342 diluted in 1× PBS to 2 μg/ml. Confocal images were taken using a Nikon A1R microscope and an Apo TIRF × 60/1.49 oil differential interference contrast (DIC) objective. To quantify GLUT4 expression on the cell surface, five images were analyzed per mouse. In each image, 25 membrane areas were randomly selected and the GLUT4 signal was quantified in the area positive for WGA staining. The average GLUT4 signal for each mouse was calculated.

### Quantitative gene expression analysis

Tissues were collected and homogenized in TRIzol (Thermo Fisher Scientific). Total RNA was isolated using the RNeasy Mini Kit (Qiagen) and RNA quantity was measured with a spectrophotometer (Nanodrop ND 1000; NanoDrop Products, Wilmington, DE). For qRT-PCR studies, 1 mg of total RNA was reversely transcribed (SuperScript III Reverse Transcriptase, Invitrogen) using oligo(dT) primers. qRT-PCR was performed using SYBR Green Master Mix (Applied Biosystems) according to the manufacturer’s instructions. Data were normalized to the expression of β_2_-microglobulin.

### RNA-Seq studies

For chronic clenbuterol studies, lean WT mice (12-week-old males) consumed clenbuterol (30 mg/l) or regular drinking water for 5 days. Subsequently, quadriceps muscles were isolated from overnight (15 h) fasted or freely fed mice. Control and SKM-Rictor-KO mice were treated in the same fashion, but gastrocnemius muscle samples were collected from fed mice only. Total RNA was prepared from frozen tissues as described above. The RNA quality of all samples was analyzed on the Agilent 2100 bioanalyzer using the RNA 6000 Nano Chip. cDNA libraries were generated using New England Biolabs Ultra II kit following the manufacturer’s instructions. Indexed libraries of good quality (RNA Integrity Number > 8) were pooled and used for sequencing on a HiSeq 2500 Illumina Sequencing System. Single end 50 bp reads were mapped to mouse genome mm10. Analysis of differentially expressed genes was performed on the Genomatix Genome Analyzer using the DESeq2 (two-tailed Wald’s test) software package. Functional enrichment and pathway analysis of differentially expressed genes was performed using Qiagen IPA software and ToppGene Suite^64^.

### Metabolomics

WT mice (10-week-old males) consumed clenbuterol (30 mg/l) or regular drinking water for 5 days. Quadriceps muscles were then collected and freeze-clamped from mice that had been fasted overnight (15 h). In addition, overnight (15 h) fasted clenbuterol and control WT mice were injected with glucose (2 g/kg i.p.) and quadriceps muscles were isolated and freeze-clamped 30 or 60 min after the i.p. glucose bolus. Sample preparation and analysis was carried out at Metabolon, Inc. (Durham, NC). In brief, sample preparation involved protein precipitation and removal with methanol, shaking, and centrifugation. The resulting extracts were profiled on an accurate mass global metabolomics platform consisting of multiple arms differing by chromatography methods and mass spectrometry ionization modes to achieve broad coverage of compounds differing by physiochemical properties such as mass, charge, chromatographic separation, and ionization behavior. The details of this platform have been described previously^65^. Metabolites were identified by automated comparison of the ion features in the experimental samples to a reference library of chemical standard entries that included retention time, molecular weight (*m*/*z*), preferred adducts, and in-source fragments, as well as associated MS spectra, and were curated by visual inspection for quality control using software developed at Metabolon^66,67^.

Statistical analysis was performed using two-way analysis of variance (ANOVA) contrasts, main and interaction effect tests as standard statistical methods used by Metabolon^68^.

### [^3^H]-BROMO uptake studies

In vivo fatty acid uptake was examined in WT male mice treated with clenbuterol (30 mg/l) or regular drinking water for 5 days. Mice were fasted overnight (15 h) and injected i.p. with 200 μl of olive oil containing 2 μCi of (R)-2-bromopalmitic acid [9,10-^3^H] ([^3^H]-BROMO), a partially metabolizable long-chain fatty acid tracer (American Radiolabeled Chemicals, St. Louis, MO). Tissues (~150 mg) were collected 1 h later and solubilized in 1 ml of Biosol (National Diagnostics, Charlotte, NC) for 3 h in a 50 °C water bath, followed by another 1 h incubation in the presence of 0.3 ml 30% hydrogen peroxide. Samples were mixed with 10 ml of scintillation solution (Bioscint, National Diagnostics, Charlotte, NC) and counted using a Tri-Carb 2910 TR liquid scintillation analyzer (Perkin Elmer, Waltham, MA). Data were normalized to mg of tissue.

### Stable isotope ([U-^13^C]-palmitate) studies

Lean WT mice (12-week-old males) consumed clenbuterol water (30 mg/l) or regular drinking water for 5 days. On the day of the experiment, overnight (15 h) fasted mice were injected with [U-^13^C]-palmitate (50 μg/kg i.p.). The injection solution was prepared as follows: [U-^13^C]-palmitate powder was dissolved in 50% ethanol and further diluted in sterile 0.9% saline containing bovine serum albumin (BSA) to the final concentration (2.5 mg/ml [U-^13^C]-palmitate, 5% ethanol, 10% BSA). BSA coupling of palmitate was achieved by shaking the incubation solution overnight at 37 °C. Mice were killed 25 min after the injection and isolated tissues were snap-frozen in liquid nitrogen for metabolite extraction.

### Acylcarnitine and stable isotope enrichment UPLC-Quadrupole Time of Flight Mass Spectrometer (QTOFMS) analysis

Quadriceps muscles were homogenized in 14 µl/mg tissue of 3 : 4 water : methanol using a Precellys tissue homogenizer (setting: shaking at 6500 r.p.m. twice for 30 s). Chloroform containing 0.5 µM C17 ceramide (d18:1/17:0, Avanti Polar Lipids) was added to each sample at a ratio of 16 µl/mg tissue. The organic layer was transferred to a glass tube and dried under a gentle stream of nitrogen. Samples were reconstituted in isopropanol/methanol/chloroform (4/2/1) and 5 μl was injected on to a Waters ACQUITY ultra-performance liquid chromatography (UPLC) CSH C18 1.7 μm column (2.1 × 100 mm) maintained at 55 °C. The mobile phase, flow rate, and gradient conditions were as previously described^69^. UPLC-QTOFMS analysis was performed in a Waters SYNAPT G2-Si in positive and negative electrospray ionization modes. Data were analyzed using Targetlynx XS and the percentage of neutral isotope was calculated as a peak area ratio of complete ^13^C-labeled metabolite/^12^C-labeled metabolite × 100%.

### Western blotting experiments

Tissues were isolated quickly, frozen in liquid nitrogen, and stored at −80 °C until use. For western blotting studies, frozen tissues were homogenized in ice-cold RIPA buffer (Sigma Aldrich) and protein concentrations were determined using a BCA protein assay (Thermo Fisher Scientific). Protein extracts were separated on NuPAGE 4–12% Bis-Tris or, for high molecular mass protein targets, on 3–8% Tris-Acetate gels (Thermo Fisher Scientific) and blotted onto nitrocellulose membranes (Bio-Rad). Membranes were blocked for 1 h at room temperature in TBS-T (0.1%) containing 5% BSA. Membranes were then incubated overnight with primary antibodies at 4 °C. Following three washing steps in TBS-T (0.1%), membranes were incubated with horseradish peroxidase-conjugated secondary antibodies for 1 h at room temperature. After thorough washing, proteins were visualized with SuperSignal West Dura Extended Duration Substrate (Thermo Fisher Scientific) on the c600 Imaging System (Azure Biosystems). Immunoreactive bands were quantified using Image J Software (NIH). Uncropped images are provided in the Source Data file.

### Statistics

Data are expressed as mean ± SEM for the indicated number of observations. Data were assessed for statistical significance by two-way ANOVA tests, followed by the indicated post hoc tests, or by using a two-tailed unpaired Student’s *t*-test, as appropriate. A *p*-value of <0.05 was considered statistically significant. The specific statistical tests that were used are indicated in the figure legends. Precise *p*-values are provided in the Source Data File.

### Reporting summary

Further information on research design is available in the Nature Research Reporting Summary linked to this article.

## Supplementary information

Supplementary Information

Description of Additional Supplementary Files

Supplementary Data 1

Reporting Summary

## Data Availability

Source data are provided as a Source Data file. Levels of all by Metabolon detected metabolites are provided as Supplementary Data 1 and raw data can be downloaded from MetaboLights under https://www.ebi.ac.uk/metabolights/MTBLS467 (study number: MTBLS467). The raw RNA-seq data can be downloaded from the NCBI Sequence Read Archive under reference number RNA seq-PRJNA756816 [https://www.ncbi.nlm.nih.gov/bioproject/PRJNA756816]. Source data are provided with this paper.

## Source data

Source Data
