# Supplementary material for: Clenbuterol exerts antidiabetic activity through metabolic reprogramming of skeletal muscle cells
Source: Nat Commun. 2022 Jan 10;13:22. doi: 10.1038/s41467-021-27540-w (PMC8748640; doi:10.1038/s41467-021-27540-w)
Supplement: Supplementary file 1 — Supplementary Information [file 41467_2021_27540_MOESM1_ESM.pdf]

Supplemental Information

Clenbuterol exerts antidiabetic activity through metabolic reprogramming of skeletal muscle cells

Jaroslawna Meister, Derek B. J. Bone, Jonas R. Knudsen, Luiz F. Barella, Thomas J. Velenosi, Dmitry Akhmedov, Regina J. Lee, Amanda H. Cohen, Oksana Gavrilova, Yinghong Cui, Gerard Karsenty, Min Chen, Lee S. Weinstein, Maximilian Kleinert, Rebecca Berdeaux, Thomas E. Jensen, Erik A. Richter, and Jürgen Wess

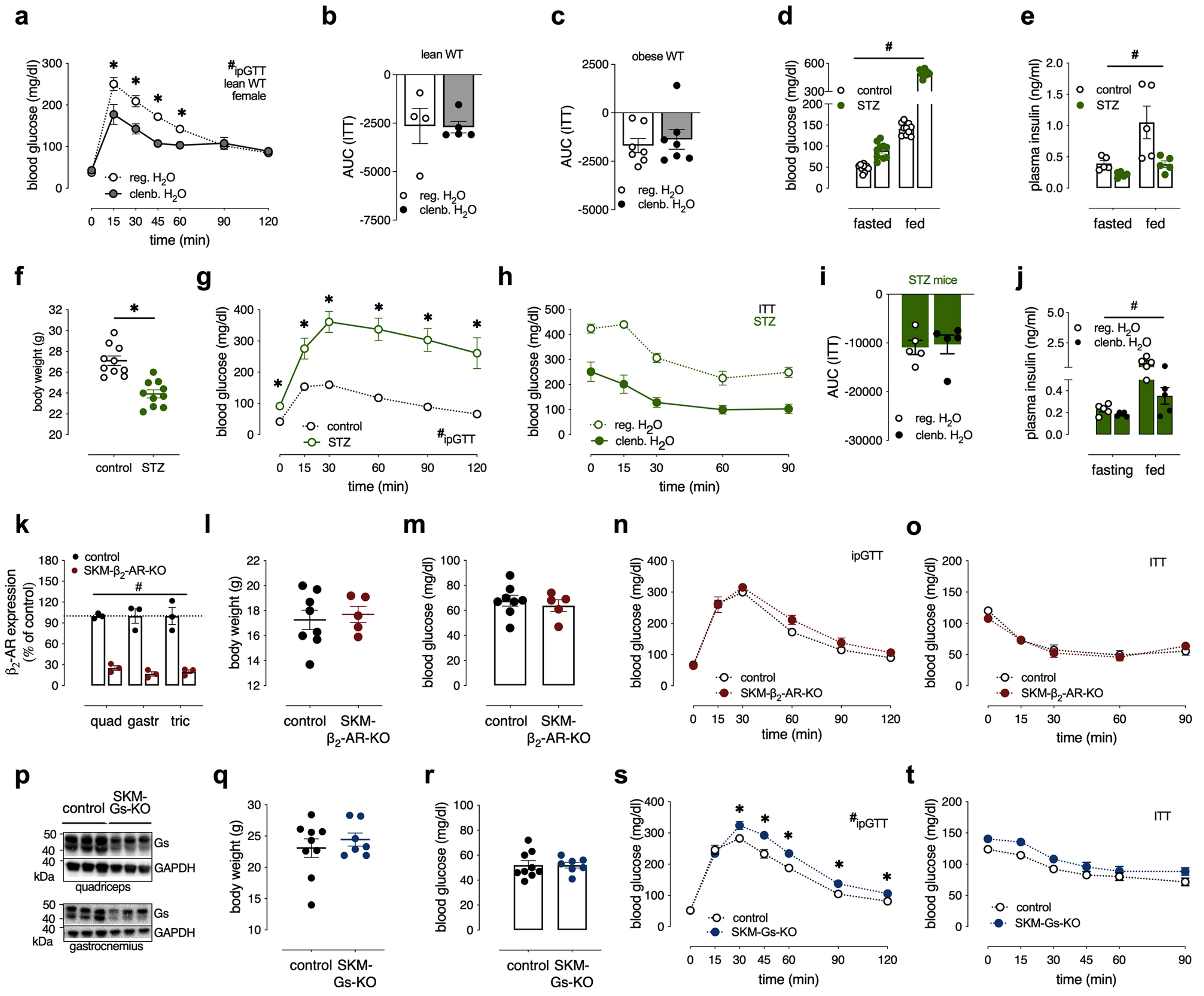

Supplementary Fig. 1. Metabolic studies with WT, SKM-β_2-AR-KO, and SKM-Gs-KO mice. All studies were carried out with male mice (8-24 weeks old), except for (**a**).

(a) Glucose tolerance test (GTT, 2 g/kg glucose i.p.) with lean female WT mice treated with clenbuterol water (clenb. H_2O) or vehicle (reg. H_2O) for 5 days (n=7/group). *p<0.05, unpaired two-tailed Student's t-test, #p<0.05, repeated measure two-way ANOVA, clenbuterol effect.

(b) Area under the curve (AUC) for insulin tolerance test (ITT) presented in Fig. 1b. AUC was calculated using the initial blood glucose value as baseline. Lean WT mice (fasted for 4 hr) injected i.p. with insulin (0.75 IU/kg) (n=4 on reg. H_2O, n=5 on clenb. H_2O)

(c) AUC for ITT presented in Fig. 1e. AUC was calculated using the initial blood glucose value as baseline. HFD-induced obese WT mice (fasted for 4 hr) injected i.p. with insulin (0.75 IU/kg) (n=7/group).

(d) Fasted and fed blood glucose levels in WT mice treated with streptozotocin (STZ) or vehicle (n=10/group). $^{\#}p<0.05$ for STZ effect by two-way ANOVA.
(e) Fasted and fed plasma insulin levels in WT mice treated with STZ or vehicle (n=5/group). $^{\#}p<0.05$ for STZ effect (two-way ANOVA).
(f) Body weight of WT mice treated with STZ or vehicle (n=10/group). *p<0.05, unpaired two-tailed Student's t-test.
(g) GTT with WT mice treated with STZ or vehicle (n=5/group). *p<0.05, unpaired two-tailed Student's t-test, #p<0.05, repeated measure two-way ANOVA, STZ effect.
(h) ITT with streptozotocin (STZ)-induced diabetic WT mice treated with clenbuterol or regular water for five days and injected with insulin (0.75 IU/kg i.p.) after 4 hr fasting (n=5/group).
(i) AUC for ITT presented in panel (**h**). AUC was calculated using the initial blood glucose value as baseline (n=5/group).
(j) Fasting and fed plasma insulin levels in STZ WT mice (n=5/group). $^{\#}p<0.05$ for clenbuterol effect (two-way ANOVA).
(k) β_2-AR gene expression levels (determined by qRT-PCR) in various SKM tissues (quadriceps, gastrocnemius and triceps) in lean control and SKM-β_2-AR-KO mice (n=3/group). $^{\#}p<0.05$, two-way ANOVA followed by Sidak's posthoc test.
(l) Body weight of lean control (n=8) and SKM-β_2-AR-KO mice (n=5).
(m) Fasting blood glucose levels in lean control (n=8) and SKM-β_2-AR-KO mice (n=5).
(n) GTT (2 g/kg glucose i.p.) carried out with overnight-fasted control (n=12) and SKM-β_2-AR-KO mice (n=7).
(o) Insulin tolerance test (ITT, 0.75 IU/kg insulin) performed with 4 hr-fasted control (n=14) and SKM-β_2-AR-KO mice (n=6).
(p) Immunoblotting analysis of Ga_s protein expression levels in SKM tissues (quadriceps and gastrocnemius) of lean control and SKM-Gs-KO mice (n=3/group).
(q) Body weight of lean control (n=9) and SKM-Gs-KO mice (n=7).
(r) Fasting blood glucose levels in control (n=9) and SKM-Gs-KO mice (n=7).
(s) GTT (2 g/kg glucose i.p.) with overnight-fasted control (n=9) and SKM-Gs-KO mice (n=7). *p<0.05, unpaired two-tailed Student's t-test, #p<0.05, repeated measure two-way ANOVA, genotype effect.
(t) Insulin tolerance test (ITT, 0.75 IU/kg insulin i.p.) with 4 h-fasted control (n=9) and SKM-Gs-KO mice (n=7).

Data are presented a means ± s.e.m. Source data are provided as a Source Data file.

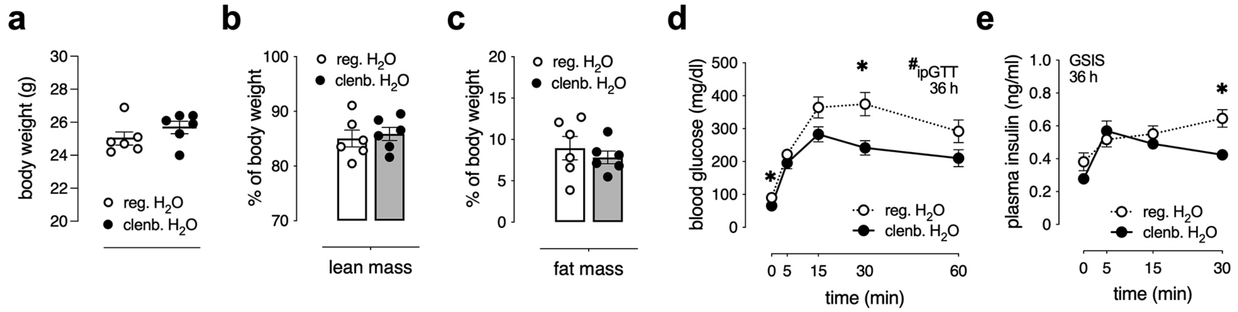

Supplementary Fig. 2. Metabolic studies with WT mice on clenbuterol water. All studies were carried out with male mice that were at least 8 weeks old.

(**a-c**) Body weight and body composition of WT mice following treatment with clenbuterol or regular drinking water for 5 days (n=6/group).

(**d, e**) Blood glucose (**d**) and plasma insulin levels (**e**). Lean overnight-fasted WT mice were injected with glucose (2 g/kg i.p.), following treatment with clenbuterol or regular drinking water for 36 hr (n=8/group). *$p<0.05$, unpaired two-tailed Student's t-test, #$p<0.05$, repeated measure two-way ANOVA, clenbuterol effect.

Data are presented as means ± s.e.m. Source data are provided as a Source Data file.

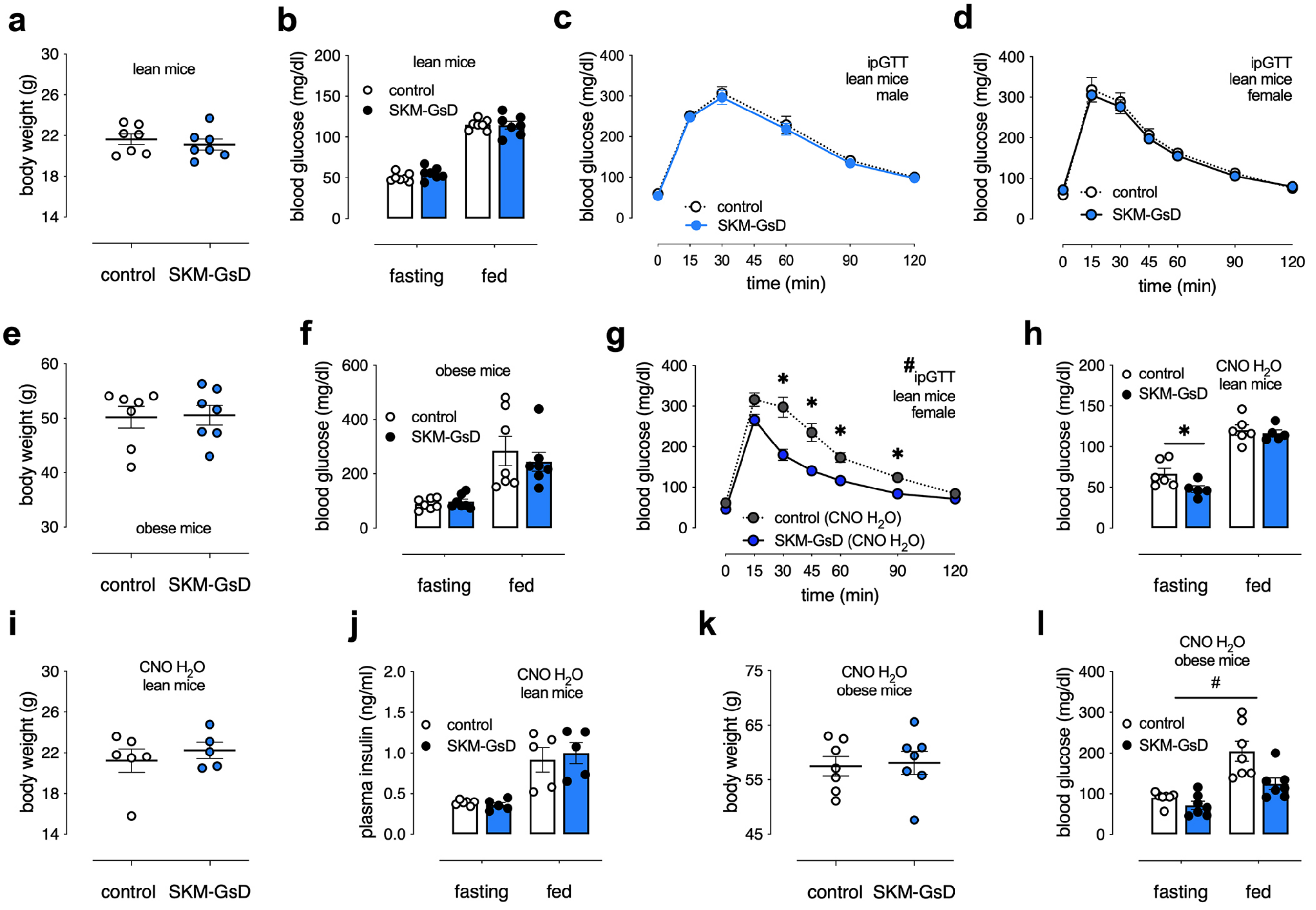

Supplementary Fig. 3. Metabolic characterization of SKM-GsD mice. All studies were carried out with male mice (except for (**d**) ang (**g**)) that were at least 8 weeks old.

(a) Body weight of lean control and SKM-GsD mice in the absence of CNO (n=7/group).

(b) Fasting and fed blood glucose levels in control and SKM-GsD mice in the absence of CNO (n=7/group).

(c) Glucose tolerance test (GTT, 2 g/kg glucose i.p.) carried out with overnight-fasted male control and SKM-GsD mice in the absence of CNO (n=7/group).

(d) GTT (2 g/kg glucose i.p.) carried out with overnight-fasted female control (n=5) and SKM-GsD mice (n=10) in the absence of CNO.

(e) Body weight of HFD-induced obese control and SKM-GsD mice (8 weeks on HFD) in the absence of CNO (n=7/group).

(f) Fasting and fed blood glucose levels in HFD-induced obese control and SKM-GsD mice in absence of CNO (n=7/group).

(g) GTT (2 g/kg glucose i.p.) carried out with overnight-fasted female control (n=5) and SKM-GsD mice (n=10) maintained on CNO (250 mg/l) drinking water for one week. $*p<0.05$, unpaired two-tailed Student's t-test, $\#p< 0.05$, repeated measure two-way ANOVA, genotype effect.

(h) Fasting and fed blood glucose levels in lean control (n=6) and SKM-GsD mice (n=5) on CNO drinking water for one week. $*p<0.05$, unpaired two-tailed Student's t-test.

(i) Body weight of lean control (n=6) and SKM-GsD mice (n=5) maintained on CNO (250 mg/l) drinking water for one week.
(j) Fasting and fed plasma insulin levels in lean control (n=6) and SKM-GsD mice (n=5) maintained on CNO drinking water for one week.
(k) Body weight of HFD-induced obese control and SKM-GsD mice maintained on CNO drinking water for one week (n=7/group).
(l) Fasting and fed blood glucose levels in HFD-induced obese control and SKM-GsD mice maintained on CNO drinking water for one week (n=7/group). $^{\#}p<0.05$ for genotype effect (two-way ANOVA).

Data are presented as means ± s.e.m. Source data are provided as a Source Data file.

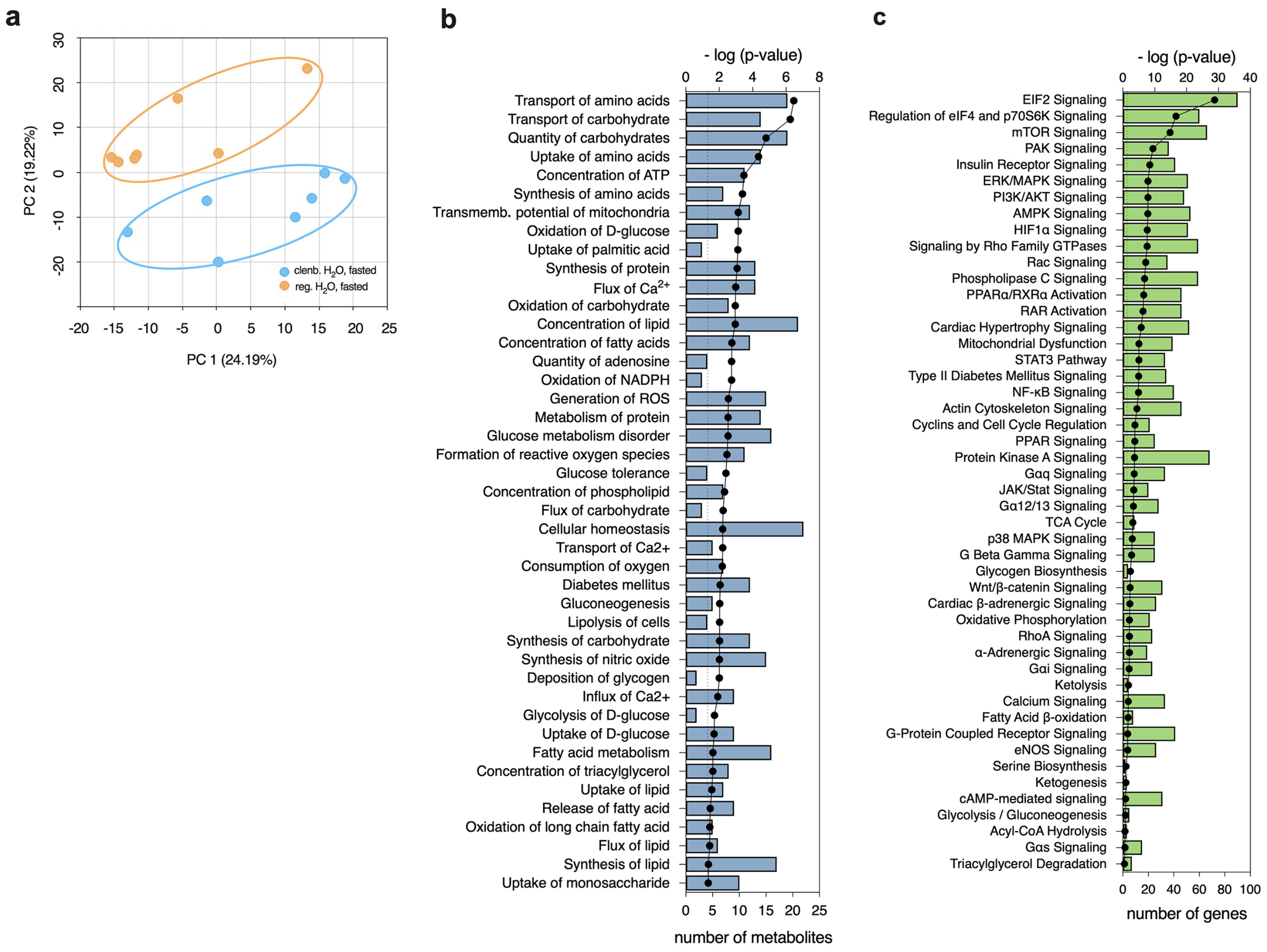

Supplementary Fig. 4. Pathway analysis of metabolomic and RNA-Seq studies with WT mice on clenbuterol drinking water. Prior to metabolomics and gene expression studies, lean WT mice consumed drinking water containing clenbuterol (30 mg/l; clenb. H_2O) or regular drinking water (reg. H_2O) for 5 days. All studies were carried out with male mice that were 12 weeks old.

(a) Principal component analysis based on detected metabolite levels in quadriceps muscle of fasted WT mice (n=7/group) consuming clenbuterol versus regular drinking water for 5 days.

(b) Pathway analysis of significantly different metabolites in quadriceps muscle of fasted WT mice (n=7/group) treated with clenbuterol compared to regular water treated mice, determined via Ingenuity Pathway Analyzer (Qiagen).

(c) Enrichment of signaling pathways of differentially expressed genes in quadriceps muscle of clenbuterol water treated mice compared to regular water treated mice. Differential gene expression was determined in SKM samples from overnight fasted (15 hr) and freely fed mice using DESeq2 via Genomatix Genome Analyzer. Pathway enrichment analysis was performed with genes that were differentially expressed in both, fasted and fed mice (total of 2817 common genes, adjusted p-value <0.05, two-tailed Wald-Test (DESeq2), n=5/group) using Ingenuity Pathway Analyzer (Qiagen).

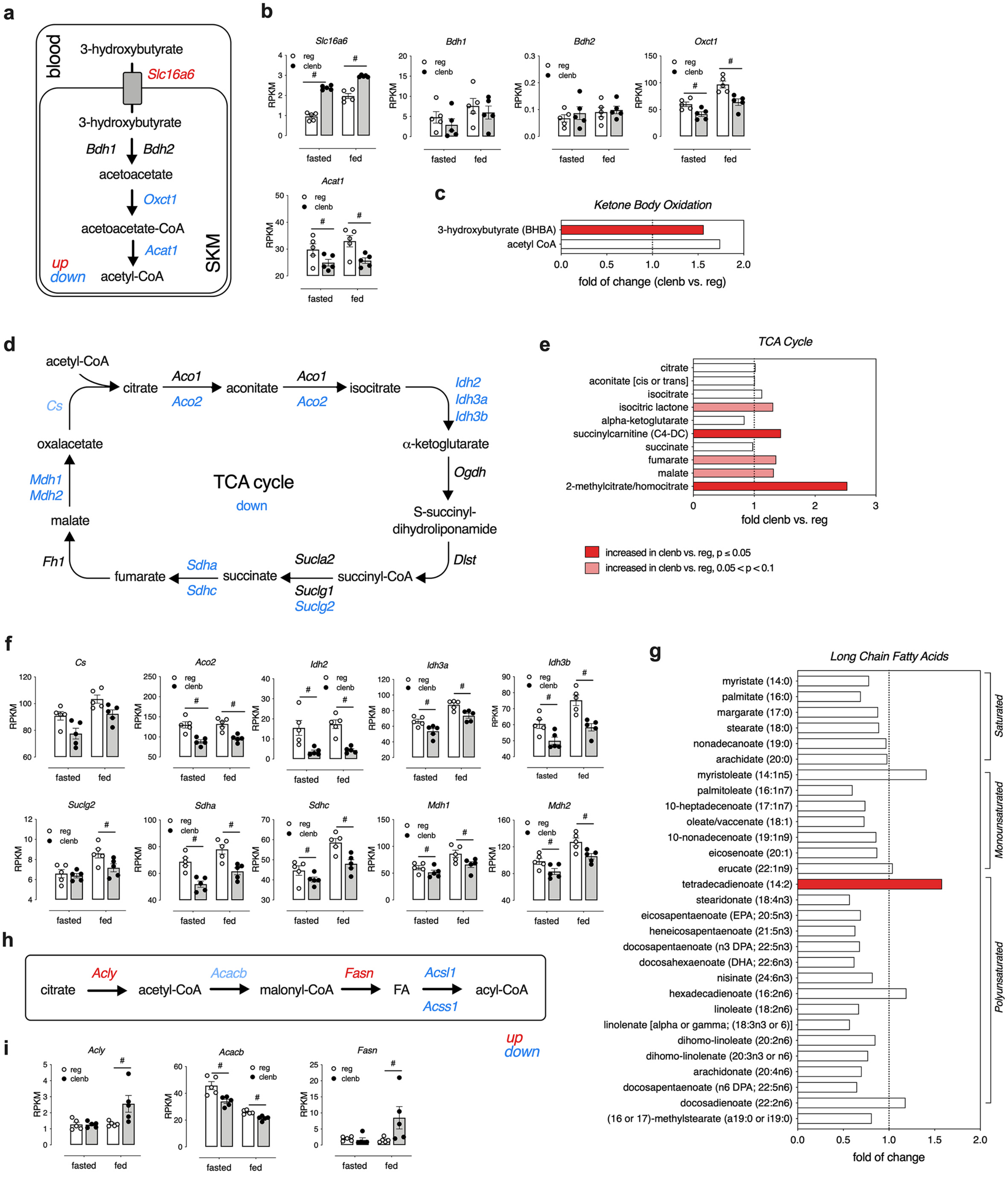

a
blood
3-hydroxybutyrate
Slc16a6
3-hydroxybutyrate
Bdh1
Bdh2
acetoacetate
Oxct1
acetoacetate-CoA
Acat1
up
down
acetyl-CoA
SKM
b
Slc16a6
Bdh1
Bdh2
Oxct1
Acat1
reg
clenb
RPKM
fasted
fed
#
c
Ketone Body Oxidation
3-hydroxybutyrate (BHBA)
acetyl CoA
0.0
0.5
1.0
1.5
2.0
fold of change (clenb vs. reg)
d
acetyl-CoA
citrate
Aco1
Aco2
aconitate
isocitrate
Idh2
Idh3a
Idh3b
Cs
oxalacetate
α-ketoglutarate
Ogdh
TCA cycle
down
Mdh1
Mdh2
S-succinyl-dihydroliponamide
malate
Fh1
fumarate
Sdha
Sdhc
succinate
Sucla2
Suclg1
Suclg2
succinyl-CoA
Dlst
e
TCA Cycle
citrate
aconitate [cis or trans]
isocitrate
isocitric lactone
alpha-ketoglutarate
succinylcarnitine (C4-DC)
succinate
fumarate
malate
2-methylcitrate/homocitrate
0
1
2
3
fold clenb vs. reg
increased in clenb vs. reg, p ≤ 0.05
increased in clenb vs. reg, 0.05 < p < 0.1
f
Cs
Aco2
Idh2
Idh3a
Idh3b
Suclg2
Sdha
Sdhc
Mdh1
Mdh2
reg
clenb
RPKM
fasted
fed
#
g
Long Chain Fatty Acids
myristate (14:0)
palmitate (16:0)
margarate (17:0)
stearate (18:0)
nonadecanoate (19:0)
arachidate (20:0)
myristoleate (14:1n5)
palmitoleate (16:1n7)
10-heptadecenoate (17:1n7)
oleate/vaccenate (18:1)
10-nonadecenoate (19:1n9)
eicosenoate (20:1)
erucate (22:1n9)
tetradecadienoate (14:2)
stearidonate (18:4n3)
eicosapentaenoate (EPA; 20:5n3)
heneicosapentaenoate (21:5n3)
docosapentaenoate (n3 DPA; 22:5n3)
docosahexaenoate (DHA; 22:6n3)
nisinate (24:6n3)
hexadecadienoate (16:2n6)
linoleate (18:2n6)
linolenate [alpha or gamma; (18:3n3 or 6)]
dihomo-linoleate (20:2n6)
dihomo-linolenate (20:3n3 or n6)
arachidonate (20:4n6)
docosapentaenoate (n6 DPA; 22:5n6)
docosadienoate (22:2n6)
(16 or 17)-methylstearate (a19:0 or i19:0)
Saturated
Monounsaturated
Polyunsaturated
0.0
0.5
1.0
1.5
2.0
fold of change
h
citrate
Acly
acetyl-CoA
Acacb
malonyl-CoA
Fasn
FA
Acsl1
Acss1
acyl-CoA
up
down
i
Acly
Acacb
Fasn
reg
clenb
RPKM
fasted
fed
#

Supplementary Fig. 5. Changes in SKM ketone body oxidation, TCA cycle metabolites and long chain fatty acids following chronic clenbuterol treatment of WT mice. All studies were carried out with male mice maintained on regular chow (age: ~12 weeks).

(a) Graphic representation of differentially expressed genes within the ketone body oxidation pathway in SKM (quadriceps muscle). Prior to gene expression analyses, lean WT mice were maintained on regular drinking water or clenbuterol water for 5 days. Red and blue labeled genes indicate significant up- or down-regulation, respectively.

(b) Gene expression levels (RPKM) of SKM transcripts involved in the ketone body oxidation pathway. RNA-Seq data were analyzed using the Genomatix Genome Analyzer platform. #Significantly regulated (two-tailed Wald test (DESeq2), adjusted p-value <0.05) (n=5/group).

(c) Fold change of 3-hydroxybutyrate and acetyl-CoA levels in SKM of overnight fasted WT mice maintained on clenbuterol vs. regular drinking water for 5 days, determined by metabolomics (n=7/group). The red bar indicates significant upregulation (two-way ANOVA contrasts); also see insert below panel (**e**) for color code).

(d) Graphic representation of differentially expressed genes within the TCA cycle pathway in SKM of overnight fasted WT mice maintained on regular drinking water vs. clenbuterol water. Blue labeled genes indicate significant downregulation.

(e) Fold change of TCA cycle metabolites in SKM of overnight fasted WT mice maintained on clenbuterol water vs. regular drinking water, determined by metabolomics (n=7/group). The red bars indicate significant upregulation (two-way ANOVA contrasts).

(f) Gene expression levels (RPKM) of SKM transcripts involved in the TCA cycle pathway. RNA-Seq expression data were analyzed using the Genomatix Genome Analyzer platform. #Significantly regulated (two-tailed Wald test (DESeq2), adjusted p-value <0.05) (n=5/group).

(g) Fold change of levels of long chain fatty acids present in SKM of overnight fasted WT mice maintained on clenbuterol water vs. regular drinking water, determined by metabolomics (n=7/group). The red bar indicates significant upregulation (two-way ANOVA contrasts; also see insert below panel (**e**) for color code).

(h) Graphic representation of differentially expressed genes within the fatty acid synthesis pathway in SKM of WT mice maintained on clenbuterol water vs. regular drinking water. Red and blue labeled genes indicate significant up- or down-regulation, respectively.

(i) Gene expression levels (RPKM) of SKM transcripts involved in the fatty acid synthesis pathway. RNA-Seq expression data were analyzed using Genomatix Genome Analyzer platform. #Significantly regulated (two-tailed Wald test (DESeq2), adjusted p-value <0.05) (n=5/group).

Data are presented as means ± s.e.m. (**b, f, i**). Source data are provided as a Source Data file.

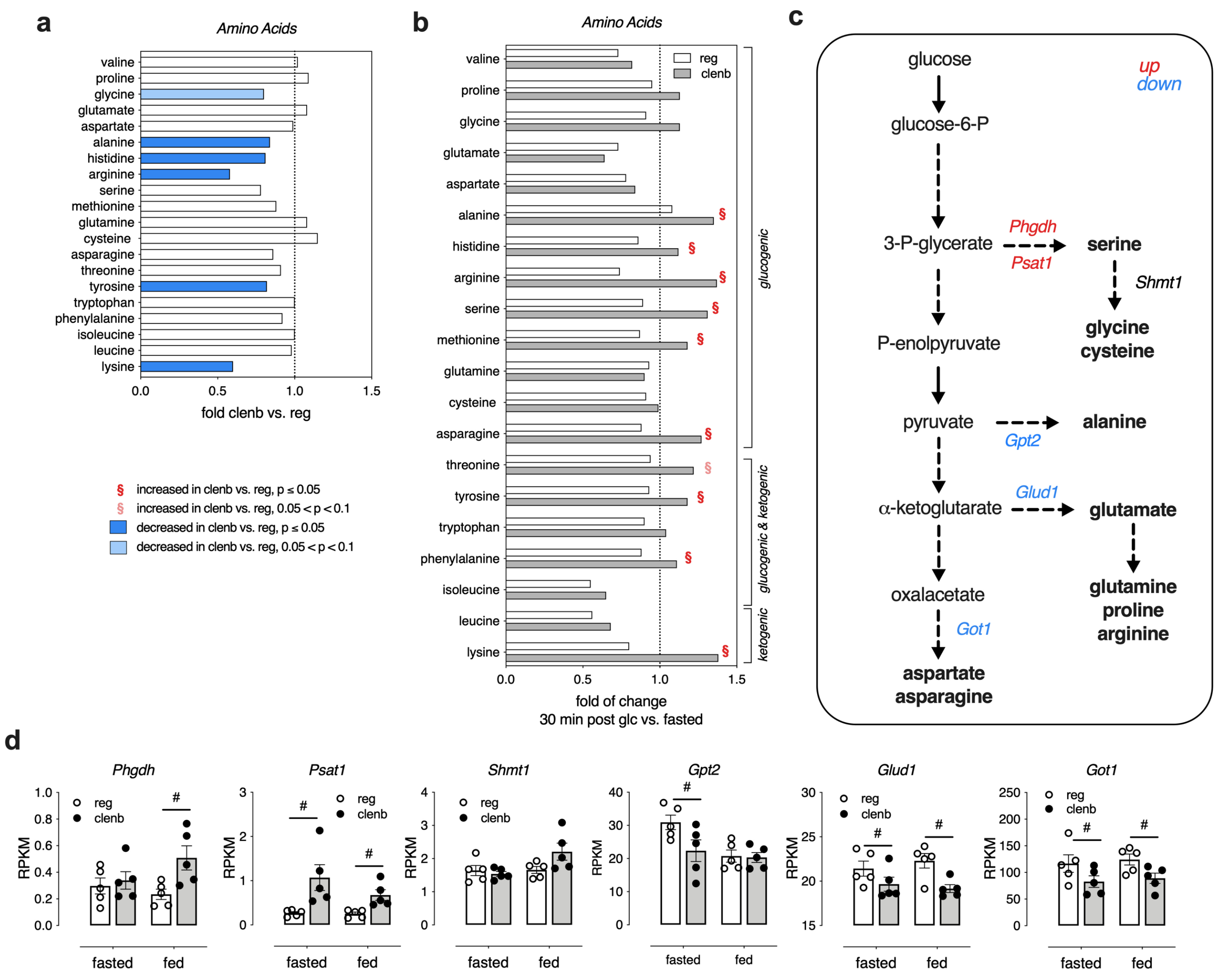

Supplementary Fig. 6. Changes in SKM amino acid metabolism following chronic clenbuterol treatment of WT mice. All studies were carried out with male mice maintained on regular chow (age: ~12 weeks).

(a) Fold change of amino acid levels in SKM (quadriceps muscle) of WT mice. Metabolomics studies were carried out with WT mice (after a 15hr fast, n=7/group) maintained on regular drinking water or clenbuterol water for 5 days. Blue bars indicate significant downregulation (two-way ANOVA contrasts). As indicated in the insert below panel (**a**), the color code represents a significant up- or down-regulation (use of two-way ANOVA contrast) of metabolites (red or blue, respectively). §Significant interaction effect between clenbuterol and glucose treatment (two-way ANOVA), also see panel (**b**).

(b) Fold changes in SKM metabolites 30 min after a glucose bolus (2 g/kg i.p.) compared to the fasted state (30 min post glucose (glc) versus fasted). §p<0.05 for interaction effect between clenbuterol and glucose treatment (two-way ANOVA, see insert below panel (**a**) for color code).

(c) Scheme of glycolysis and TCA pathways generating non-essential amino acids. Red and blue labeled genes indicate significant up- or down-regulation, respectively.

(d) Expression levels (RPKM) of selected SKM transcripts involved in transamination pathways. RNA-Seq expression data were analyzed using the Genomatix Genome Analyzer platform. [#]Significantly regulated (two-tailed Wald test (DESeq2), adjusted p-value <0.05) (n=5/group). Data are presented as means ± s.e.m. Source data are provided as a Source Data file.

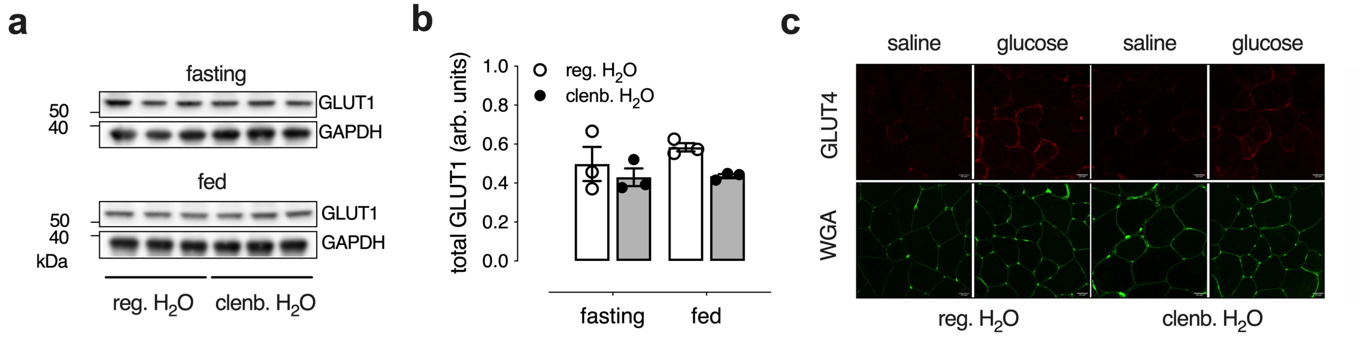

Supplementary Fig. 7. Glucose transporter protein levels in SKM following chronic clenbuterol treatment.

(a) GLUT1 protein expression in fasted and fed WT mice (males) following chronic clenbuterol or vehicle treatment 5 days (n=3/group).

(b) Quantification of the western blots shown in (**a**). Data are presented as means ± s.e.m.

(c) Representative images of GLUT4 translocation analysis (Fig. 3e). Following consumption of clenbuterol (30 mg/l) water or regular drinking water for 5 days, WT mice were fasted overnight and injected with glucose (2 g/kg i.p.) or saline. Quadriceps muscle samples were collected 15 min later. Five sections were analyzed per mouse (n=5 mice/group). Source data are provided as a Source Data file.

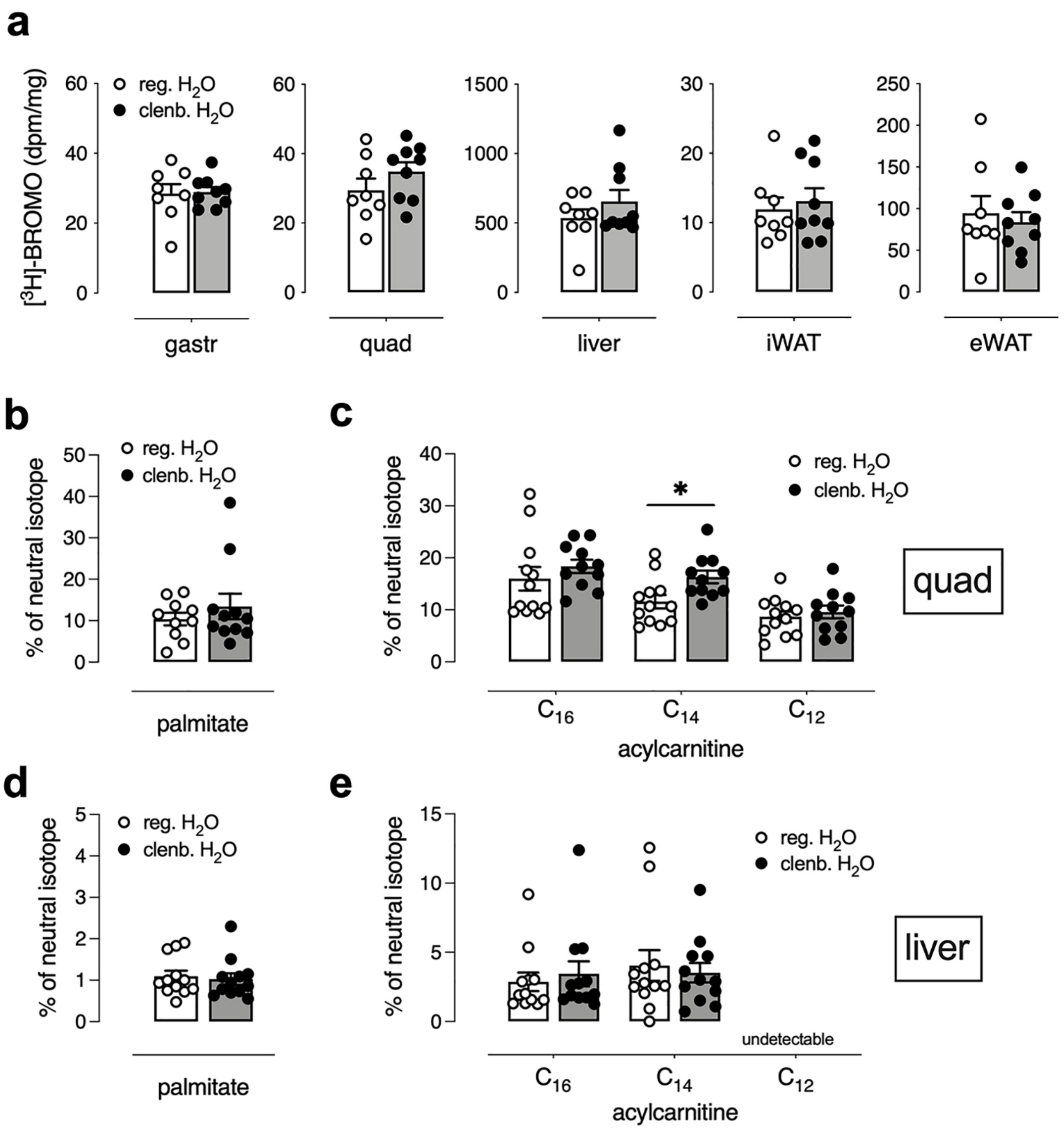

Supplementary Fig. 8. Fatty acid metabolism in SKM following chronic clenbuterol treatment. All studies were carried out with male mice that were 12-14 weeks old. Prior to isotope injection studies, lean WT mice consumed drinking water containing clenbuterol (30 mg/l; clenb. H_2O; n=8) or regular drinking water (reg. H_2O; n=9) for 5 days. Fatty acid uptake was determined in selected tissues 1 hr following the injection of [9,10-^{3}H]-(R)-2-bromopalmitate ([^{3}H]-BROMO, 2 μCi, i.p.).

(b-e) Overnight fasted mice were injected with [U-^{13}C]-palmitate (50 ug/kg, i.p.), and tissues were collected 25 min after the injection (n=12/group).

(a) Amount of ^{13}C-labeled palmitate in quadriceps muscle.
(b) Amount of selected ^{13}C-labeled acylcarnitines in quadriceps muscle.
(c) Amount of ^{13}C-labeled palmitate in liver.
(e) Amount of selected ^{13}C-labeled acylcarnitines in liver.
Data are presented as means ± s.e.m. *$p < 0.05$, unpaired two-tailed Student's t-test (neutral isotope=^{12}C).
Gastr, gastrocnemius muscle; quad., quadriceps muscle. Source data are provided as a Source Data file.

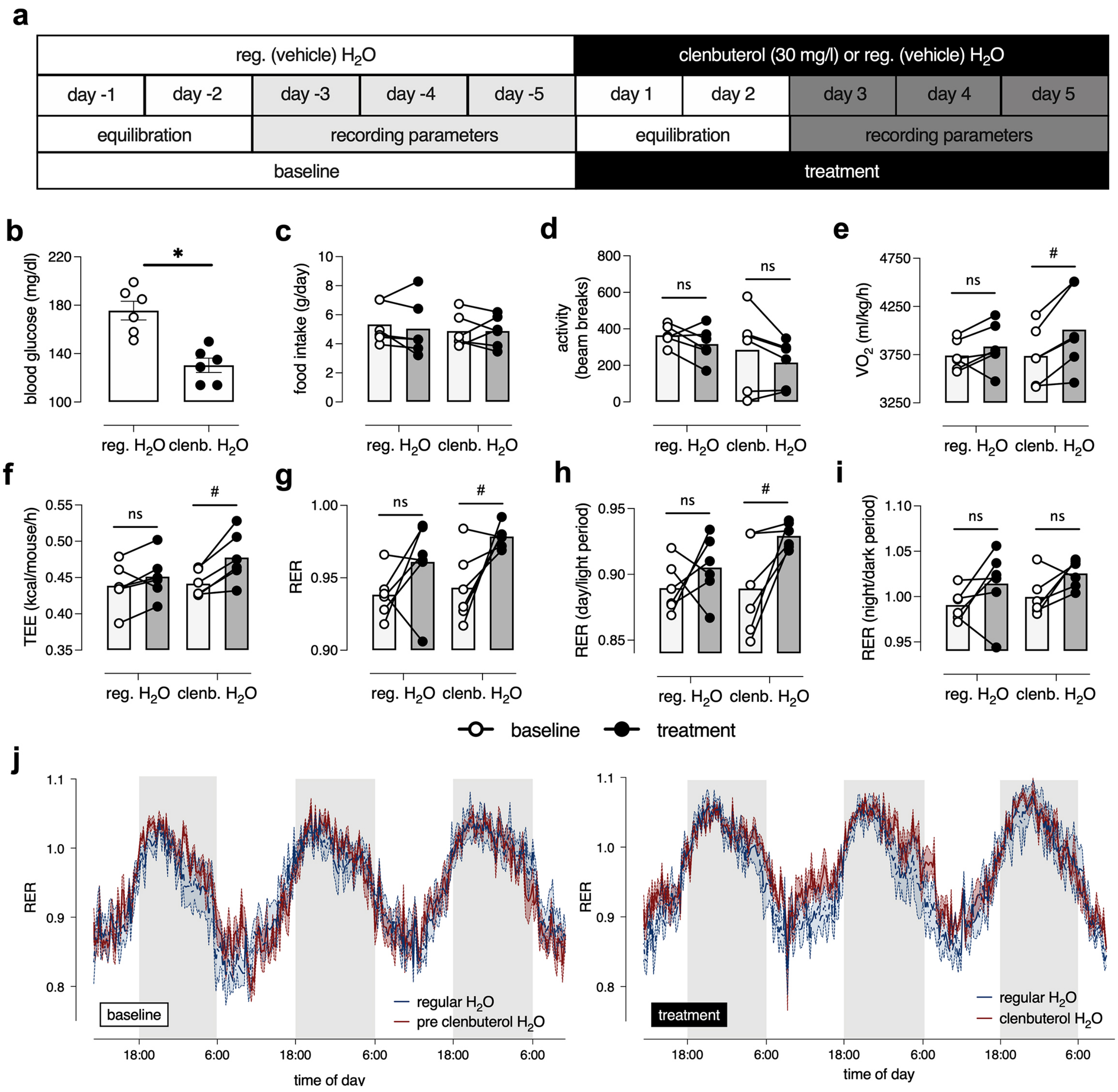

Supplementary Fig. 9. Indirect calorimetry measurements with WT mice following chronic clenbuterol treatment. All studies were carried out with male mice that were ~12 weeks old.

(a) Experimental set-up. Lean WT mice maintained on regular drinking water were placed in metabolic chambers. After a 2-day equilibration period, several metabolic parameters were monitored for 3 consecutive days (baseline). Subsequently, 6 of the mice were randomly assigned to a 5-day clenbuterol (30 mg/l) water treatment. Measurements were recorded for the 3 last days of treatment. Baseline and treatment data are represented as averages of 3 days for each mouse (n=6/group).

(b) Blood glucose levels, fed, $^*p< 0.05$, unpaired two-tailed Student's t-test

(c) Food intake measurements.
(d) Locomotor activity.
(e) Oxygen consumption (VO_2).
(f) Total energy expenditure (TEE).
(g) Respiratory exchange ratio (RER).
(h) RER for daytime (light period).
(i) RER for nighttime (dark period).
(j) RER time traces for baseline (left panel) and treatment (right panel) periods. Data are presented as means ± s.e.m.

Data are presented as means ± s.e.m. $^{\#}P < 0.05$ by repeated measure two-way ANOVA, followed by Sidak's posthoc test. Source data are provided as a Source Data file.

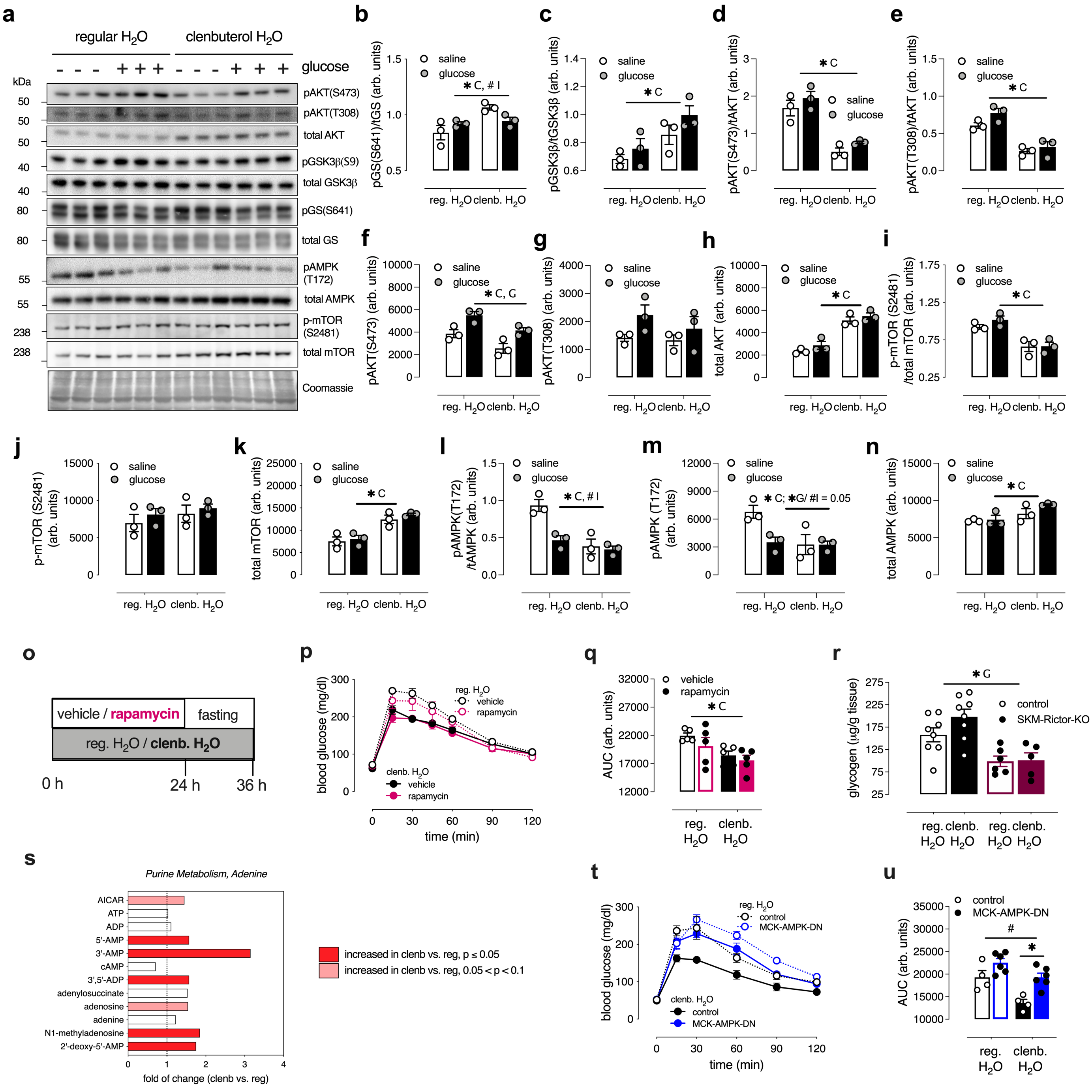

Supplementary Fig. 10. Studies involving mTORC and AMPK signaling. All studies were carried out with male mice.

(a) Western blotting studies. Following consumption of clenbuterol water (30 mg/l, clenb.) or regular drinking water (reg.) for 5 days, mice were fasted overnight and injected with glucose (2 g/kg i.p.). Quadriceps muscle samples were collected 30 min later.

(b-n) Quantification of the western blots shown in panel (**a**) (n = 3/group). *C, p<0.05 for clenbuterol effect;

*G, $p<0.05$ for glucose effect (two-way ANOVA); #I, $p<0.05$ for interaction effect between glucose and clenbuterol treatment.

(o) Experimental set-up for mTORC1 inhibition by rapamycin. Lean WT mice were randomly assigned to four treatment groups. To avoid inhibition of mTORC2, rapamycin was given for 24 hr only (10 mg/l in drinking water), while the clenbuterol treatment (30 mg/l) continued for another 12 hr for a total treatment period of 36 h.

(p) Glucose tolerance test (GTT) with overnight fasted WT mice injected with glucose (2 g/kg i.p.) following the treatment protocol illustrated in panel (**a**) (n=5/group).

(q) Area under the curve (AUC) representation of the GTT data presented in (B). *C, $p<0.05$ for clenbuterol effect (two-way ANOVA).

(r) Glycogen levels in SKM (gastrocnemius muscle) of freely fed control (n = 8) and SKM-Rictor-KO mice (n = 6) consuming regular drinking water or clenbuterol water for 5 days. *G, $p<0.05$ for genotype effect (two-way ANOVA).

(s) Fold change of adenine derivative levels in SKM of WT mice consuming clenbuterol water versus regular drinking water, following a 15-h fast, determined by metabolomics (n=7/group). See insert next to panel for color code explanation (use of two-way ANOVA contrasts for statistical significance).

(t) GTT with overnight-fasted control (n = 4) and SKM-AMPK-DN mice (n = 6) treated with reg. H_2O or clenb. H_2O for 5 days.

(u) AUC representation of the GTT data shown in (M). #$p<0.05$ for clenbuterol effect (two-way ANOVA); *$p<0.05$ (two-way ANOVA, followed by Sidak's posthoc test).

Data are presented as means ± s.e.m. Source data are provided as a Source Data file.

Supplementary Table 1. Expression analysis of selected transcripts involved in major metabolic pathways following chronic clenbuterol treatment of control and SKM-Rictor-KO mice

	Control regular H_2O		SKM-Rictor-KO regular H_2O		Control clenbuterol H_2O		SKM-Rictor-KO clenbuterol H_2O	
	RPKM (mean)	RPKM (SEM)	RPKM (mean)	RPKM (SEM)	RPKM (mean)	RPKM (SEM)	RPKM (mean)	RPKM (SEM)
**Glycolysis**								
Slc2a1	1.8	0.2	1.8	0.2	1.8	0.2	1.3	0.1
Slc2a4	104.6	2.6	96.2	5.0	93.4	3.7	85.1	2.4
Hk2	40.7	1.6	33.3	1.8	38.0	2.2	32.3	2.3
Gpi1	56.7	1.8	59.0	2.0	66.3	4.1	69.4	5.4
Pfkm	662.8	16.3	609.9	14.8	649.1	29.7	610.3	27.1
Aldoa	2177.9	58.6	2164.1	55.0	2463.3	64.8	2621.4*	97.7
Gapdh	2230.9	68.1	2366.8	61.2	2596.2*	59.5	2939.6*	116.2
Pgk1	16.9	1.0	18.3	1.2	26.1*	2.7	29.5*	2.5
Pgam2	1403.4	49.1	1596.2	95.1	1632.5	92.9	1856.1	126.1
Eno1	2.8	0.2	3.9	0.2	3.7*	0.3	4.1	0.4
Eno3	843.0	19.9	977.4	47.2	1068.6	118.2	1225.4	102.7
Pkm	226.8	3.6	221.2	6.1	231.3	9.1	231.2	9.6
Pdha1	126.2	5.9	118.8	5.4	137.0	6.3	147.4*	4.9
Pdhx	10.1	0.2	10.7	0.3	11.0	0.5	11.9	0.4
Pdk1	4.8	0.2	4.6	0.3	3.8*	0.2	3.8	0.3
Pdk2	116.5	3.0	125.2	2.8	84.1*	2.1	92.3*	2.1
Pdk4	126.5	25.5	155.8	25.3	57.0	11.9	91.1*	9.7
Ldha	413.3	11.2	423.8	21.1	502.3	38.5	520.7	33.8
Ldhb	48.5	5.4	43.1	7.0	20.6*	5.4	19.2*	3.0
Ldhd	9.8	0.5	10.9	0.8	7.5*	0.2	8.1*	0.3
**Glycogen metabolism**								
Gyg	110.6	3.2	91.9	1.9	75.9*	3.5	69.5*	1.9
Gys1	73.4	1.9	69.1	3.4	55.5*	4.0	54.2	4.2
Gsk3b	4.4	0.1	3.6	0.1	3.6*	0.2	3.3	0.1
Ppp1r1a	8.5	0.5	19.6	2.4	5.6*	0.4	12.4*	1.1
**Ketone oxidation**								
Slc16a6	2.1	0.1	1.9	0.1	1.4*	0.1	1.3*	0.1
Bdh1	9.2	1.2	7.3	1.3	9.3	3.0	7.0	1.2
Bdh2	0.1	0.0	0.1	0.0	0.1	0.0	0.1	0.0
Oxct1	59.0	3.5	65.1	2.7	45.5	4.4	54.0*	1.8
**TCA cycle**								

Cs	96.4	1.1	104.5	3.1	86.9	3.2	92.2	1.7
Aco2	225.0	7.2	232.8	10.5	161.0*	8.2	168.9*	6.5
Idh2	47.6	3.6	48.9	6.0	14.4*	3.0	16.5*	2.7
Idh3a	70.6	2.0	74.3	2.6	61.9	3.9	66.1	2.2
Idh3b	64.2	1.7	67.6	1.9	50.9*	2.3	56.5*	2.4
Suclg2	8.4	0.1	8.6	0.3	7.1	0.3	7.0	0.4
Sdha	63.4	2.0	67.6	2.1	51.0*	2.4	55.9*	2.3
Shdb	42.2	1.7	46.7	3.2	37.8	2.6	42.4	2.3
Mdh1	61.2	2.5	62.3	5.0	49.5	5.0	51.3	3.0
Mdh2	112.7	4.1	116.0	5.1	94.8	4.5	103.2	4.4
**Lipogenesis**								
Acly	1.7	0.2	4.3	2.0	2.2	0.4	1.6	0.1
Acacb	28.7	1.4	25.6	1.9	23.5	0.7	21.6	1.6
Fasn	3.2	1.1	15.9	10.4	3.9	1.1	2.7	0.7

Gene expression levels (RPKM) in gastrocnemius muscles of freely fed control and SKM-Rictor-KO mice consuming regular or clenbuterol water for 5 days (n=5/group).
*Adjusted $p<0.05$, for clenbuterol effect compared to regular water treatment in the indicated genotype (DeSeq2, two-tailed Wald test).

Supplementary Table 2. Antibodies, chemicals, assay kits, and mouse lines used in this study

Reagent or Resource	Source	Identifier
**Antibodies**		(Dilution used in parentheses)
Glycogen Synthase (15B1) Rabbit mAb	Cell Signaling Technology	3886 (1:1000)
Phospho-Glycogen Synthase (Ser641) antibody	Cell Signaling Technology	3891 (1:1000)
AMPKα antibody	Cell Signaling Technology	2532 (1:1000)
Phospho-AMPKα (Thr172) (D4D6D) Rabbit mAb	Cell Signaling Technology	50081 (1:1000)
GAPDH (14C10) Rabbit mAb (HRP Conjugate)	Cell Signaling Technology	3683 (1:1000)
Akt antibody	Cell Signaling Technology	9272 (1:1000)
Phospho-Akt (Ser473) antibody	Cell Signaling Technology	9271 (1:1000)
Phospho-Akt (Thr308) (C31E5E) Rabbit mAb	Cell Signaling Technology	2965 (1:1000)
GSK-3β (27C10) Rabbit mAb	Cell Signaling Technology	9315 (1:1000)
Phospho-GSK-3β (Ser9) antibody	Cell Signaling Technology	9336 (1:1000)
mTOR (7C10) Rabbit mAb	Cell Signaling Technology	2983 (1:1000)
Phospho-mTOR (Ser2481) antibody	Cell Signaling Technology	2974 (1:1000)
Glut1 Rabbit Antibody	Novus Biologicals	NB110-39113
GLUT4 Polyclonal Antibody	ThermoFisher Scientific	PA5-23052 (1:1000)
$G\alpha_s$-specific antibody	Kindly provided by Dr. Lee Weinstein	(1:3000)
Anti-mouse IgG, HRP-linked antibody	Cell Signaling Technology	7076 (1:3000)
Anti-rabbit IgG, HRP-linked antibody	Cell Signaling Technology	7074 (1:3000)
**Chemicals**		
Tamoxifen	Sigma-Aldrich	T5648
D-glucose, anhydrous, granular	Macron Fine Chemicals	4912-12
Insulin (Humulin R U-100)	Eli Lilly	
Clenbuterol	Sigma-Aldrich	C5423
cOmplete, EDTA-free Protease Inhibitor Cocktail	Sigma-Aldrich (Roche)	11873580001

Phospho-STOP	Sigma-Aldrich (Roche)	04906837001
Streptozotocin	Sigma-Aldrich	S0130
Corn oil	Sigma-Aldrich	C8267
ECL western blotting substrate	Pierce	32106
Trizol	Invitrogen	15596018
SuperScriptTM III First-Strand Synthesis SuperMix	Invitrogen	18080400
Power SYBR Green PCR Master Mix	Applied Biosystems	4367659
RIPA buffer	Sigma-Aldrich	R0278
NuPAGE™ 4-12% Bis-Tris Gel	Invitrogen	NP0336BOX
NuPAGE™ 3-8% Tris-Acetate Gel	Invitrogen	EA03785BOX
Tris Buffered Saline (TBS), 10X, pH 7.4	Quality Biological	351-086-101
NuPAGE™ MOPS SDS Running Buffer (20X)	Invitrogen	NP0001
NuPAGE™ Tris-Acetate SDS Running Buffer (20X)	Invitrogen	LA0041
Deoxy-D-glucose, 2-[1-14C]-	Perkin Elmer	NEC495
Glucose, D-[3-3H]-	Perkin Elmer	NET331C
Sodium Palmitate (U-13C16)	Cambridge Isotope Laboratories, Inc.	CLM-6059-PK
R-2-Bromopalmitic acid [9,10-3H]	American Radiolabeled Chemicals, Inc.	ART 1270-50 μCi
**Commercial Assays**		
Ultra Sensitive Mouse Insulin ELISA Kit	Crystal Chem	90080
Glycogen Assay Kit	Cayman Chemical	700480
BCA protein assay	Pierce	23225
RNeasy mini kit	Qiagen	74104
Agilent RNA 600 nano kit	Agilent Technologies	5067-1511
Agilent High Sensitivity DNA Kit	Agilent Technologies	5067-4626
NEBNext® Ultra™ II RNA Library Prep with Sample Purification Beads	New England Biolabs	E7775S
NEBNext® Poly(A) mRNA Magnetic Isolation Module	New England Biolabs	E7490

NEBNext® Multiplex Oligos for Illumina® (Index Primers Set 1 and Set 2)	New England Biolabs	E7335, E7500
**Experimental Models: Organisms/Strains**		
C57BL/6N mice	Taconic	C57BL/6NTac, B6-M, DIO-B6-M
Floxed *Adrb2*	Mice provided by Dr. Gerard Karsenty	REF[1]
Floxed *Gnas (Gα$_s$)*	Mice provided by Dr. Lee Weinstein	REF[2]
Rictor$^{flox/flox}$, HSA-Cre	Mice provided by Prof. Markus Rüegg	REF[3]
FloxSTOP-Gs-DREADD knock-in	Mice provided by Dr. Rebecca Berdeaux	REF[4]
HSA-Cre-ER^{T2}	Mice provided by Dr. Daniel Metzger	REF[5]
MCK-AMPK-DN	Mice provided by Dr. Henriette van Praag	REF[6]

1 Shi, Y. *et al*. Dissociation of the neuronal regulation of bone mass and energy metabolism by leptin in vivo. *Proc Natl Acad Sci U S A* **105**, 20529-20533, doi:10.1073/pnas.0808701106 (2008).

2 Chen, M. *et al*. G(s)alpha deficiency in skeletal muscle leads to reduced muscle mass, fiber-type switching, and glucose intolerance without insulin resistance or deficiency. *Am J Physiol Cell Physiol* **296**, C930-940, doi:10.1152/ajpcell.00443.2008 (2009).

3 Kleinert, M. *et al*. mTORC2 and AMPK differentially regulate muscle triglyceride content via Perilipin 3. *Mol Metab* **5**, 646-655, doi:10.1016/j.molmet.2016.06.007 (2016).

4 Akhmedov, D. *et al*. Gs-DREADD Knock-In Mice for Tissue-Specific, Temporal Stimulation of Cyclic AMP Signaling. *Mol Cell Biol* **37**, doi:10.1128/MCB.00584-16 (2017).

5 Schuler, M., Ali, F., Metzger, E., Chambon, P. & Metzger, D. Temporally controlled targeted somatic mutagenesis in skeletal muscles of the mouse. *Genesis (New York, N.Y. : 2000)* **41**, 165-170, doi:10.1002/gene.20107 (2005).

6 Mu, J., Brozinick, J. T., Jr., Valladares, O., Bucan, M. & Birnbaum, M. J. A role for AMP-activated protein kinase in contraction- and hypoxia-regulated glucose transport in skeletal muscle. *Mol Cell* **7**, 1085-1094 (2001).
