# Supplementary material for: Clenbuterol exerts antidiabetic activity through metabolic reprogramming of skeletal muscle cells
Source: Nat Commun. 2022 Jan 10;13:22. doi: 10.1038/s41467-021-27540-w (PMC8748640; doi:10.1038/s41467-021-27540-w)
Supplement: Supplementary file 2 — Description of Additional Supplementary Files [file 41467_2021_27540_MOESM2_ESM.pdf]

Description of Additional Supplementary Files

File Name: Supplementary Data 1

Description: Heatmap with fold changes of all detected metabolites in SKM of clenbuterol-treated mice vs. control mice
